# ZIP8 Zinc Transporter: Indispensable Role for Both Multiple-Organ Organogenesis and Hematopoiesis In Utero

**DOI:** 10.1371/journal.pone.0036055

**Published:** 2012-05-01

**Authors:** Marina Gálvez-Peralta, Lei He, Lucia F. Jorge-Nebert, Bin Wang, Marian L. Miller, Bryan L. Eppert, Scott Afton, Daniel W. Nebert

**Affiliations:** 1 Department of Environmental Health, and Center for Environmental Genetics (CEG), University of Cincinnati Medical Center, Cincinnati, Ohio, United States of America; 2 Department of Chemistry, University Cincinnati School of Arts and Sciences, Cincinnati, Ohio, United States of America; University of Illinois at Chicago, United States of America

## Abstract

Previously this laboratory characterized *Slc39a8*-encoded ZIP8 as a Zn^2+^/(HCO_3_^–^)_2_ symporter; yet, the overall physiological importance of ZIP8 at the whole-organism level remains unclear. Herein we describe the phenotype of the hypomorphic *Slc39a8(neo/neo)* mouse which has retained the neomycin-resistance gene in intron 3, hence causing significantly decreased ZIP8 mRNA and protein levels in embryo, fetus, placenta, yolk sac, and several tissues of neonates. The *Slc39a8(neo)* allele is associated with diminished zinc and iron uptake in mouse fetal fibroblast and liver-derived cultures; consequently, *Slc39a8(neo/neo)* newborns exhibit diminished zinc and iron levels in several tissues. *Slc39a8(neo/neo)* homozygotes from gestational day(**GD**)-11.5 onward are pale, growth-stunted, and die between GD18.5 and 48 h postnatally. Defects include: severely hypoplastic spleen; hypoplasia of liver, kidney, lung, and lower limbs. Histologically, *Slc39a8(neo/neo)* neonates show decreased numbers of hematopoietic islands in yolk sac and liver. Low hemoglobin, hematocrit, red cell count, serum iron, and total iron-binding capacity confirmed severe anemia. Flow cytometry of fetal liver cells revealed the erythroid series strikingly affected in the hypomorph. Zinc-dependent 5-aminolevulinic acid dehydratase, required for heme synthesis, was not different between *Slc39a8(+/+)* and *Slc39a8(neo/neo)* offspring. To demonstrate further that the mouse phenotype is due to ZIP8 deficiency, we bred *Slc39a8(+/neo)* with BAC-transgenic *BTZIP8-3* line (carrying three extra copies of the *Slc39a8* allele); this cross generated viable *Slc39a8(neo/neo)_BTZIP8-3(+/+)* pups showing none of the above-mentioned congenital defects–proving *Slc39a8(neo/neo)* causes the described phenotype. Our study demonstrates that ZIP8-mediated zinc transport plays an unappreciated critical role during in utero and neonatal growth, organ morphogenesis, and hematopoiesis.

## Introduction

The solute carrier gene (***SLC***) superfamily currently comprises at least 374 putative transporter protein-coding genes arranged in 58 gene families (http://www.bioparadigms.org/slc/menu.asp). Transported substrates include essential metals, amino acids and oligopeptides, glucose and other sugars, inorganic cations and anions [H^+^, (HCO_3_)^–^, (NH_4_)^+^, Cl^–^, Na^+^, K^+^, Ca^2+^, Mg^2+^, OH^–^, (PO_4_)^3–^, (HPO_4_)^2–^, (H_2_PO_4_)^–^, (SO_4_)^2–^, (C_2_O_4_)^2–^, (CO_3_)^2–^], bile salts, carboxylate and other organic anions, acetyl-CoA, vitamins, folate, fatty acids and lipids, biogenic amines, neurotransmitters, nucleosides, choline, thyroid hormone, and urea [1].

The mammalian *SLC39* family contains 14 members of the **z**inc- and **i**ron-related **p**rotein (**ZIP**) family, which transport essential-metal divalent cations such as zinc, iron, copper and manganese [1]; [2]; these 14 genes are very highly conserved between human and rodent. The ZIP transporter proteins appear to serve crucial roles in metal homeostasis and perhaps unappreciated important signaling pathways; for example, mutations in the human *SLC39A4* gene are responsible for acrodermatitis enteropathica, zinc-deficiency (**AEZ**) [3]–[5]. Mutations in the human *SLC39A13* gene result in a spondylocheiro dysplastic form of Ehlers-Danlos syndrome showing generalized skeletal dysplasia [6]. Endogenous functions and importance of the other twelve ZIP transporters are not yet fully understood.

Starting with genetically “sensitive” vs “resistant” inbred strains of mice and a phenotype of cadmium (**Cd**)-induced testicular necrosis, our lab launched a forward-genetics study to identify and characterize the major locus (*Cdm*) responsible for this trait [7]. By means of positional cloning, we identified *Slc39a8*, which encodes the metal transporter ZIP8 as the likely candidate for the *Cdm* locus; ZIP8 mRNA expression is robust in endothelial cells of the testicular vasculature in two Cd-sensitive mouse lines and negligible in these cells from two Cd-resistant mouse lines [8]. That the *Slc39a8* gene is indeed the *Cdm* locus was unequivocally proven–using a bacterial artificial chromosome (**BAC**)-transgenic mouse line. A 168.7-kb BAC, containing only the *Slc39a8* gene (plus 87 kb of 5′-flanking and 18 kb of 3′-flanking sequences) from a 129S6/SvEvTac (Cd-sensitive) BAC library, was inserted into the Cd-resistant C57BL/6J genome; Cd treatment produced testicular necrosis in BAC-transgenic *BTZIP8-3* mice but not in non-transgenic littermates [9].

Further studies have shown that ZIP8 functions endogenously as a Zn^2+^/(HCO_3_^–^)_2_ as well as Mn^2+^/(HCO_3_^–^)_2_ symporter–moving both ions into the cell in an electroneutral manner [10]–[12]. Moreover, Cd^2+^ is able to displace Zn^2+^, thereby entering cells that carry the functional ZIP8 transporter. A common theme in pharmacology and toxicology is that drugs and other environmental agents usurp the same receptors and transporters as those used by endogenous substrates; likewise, drugs and other environmental agents are metabolized by enzymes that normally handle endogenous substrates [13].

The next step in understanding ZIP8’s endogenous functions was to generate a knockout mouse line. In building the *Slc39a8* knockout construct [14], we inserted an *Frt*-flanked neomycin-resistance mini-gene (***neo***) into intron 3, combined with *loxP* sites in introns 3 and 6. Retention of the *neo* gene in intron 3 leads to an intriguing hypomorph. Its phenotype is reported herein–a combination of stunted growth, severe anemia, dysregulation of hematopoiesis, multiple organs failing to develop normally in utero, and neonatal lethality.

## Results

### Characterization of *Slc39a8(neo/neo)* Mice

**Figure 1:**
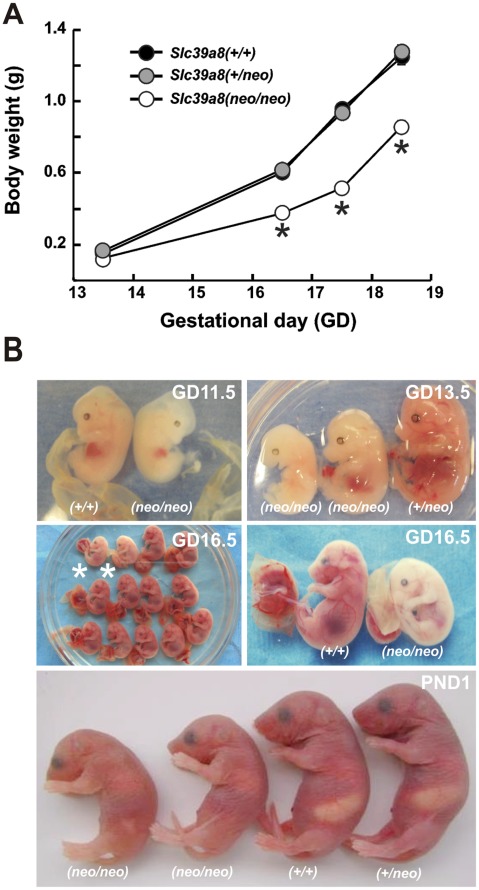
Characterization of *Slc39a8(neo/neo)* phenotype. (**A**) Body weight of embryos and fetuses of the three genotypes–from GD13.5 through GD18.5. N = 15–20 per genotype. * *P*<0.001, compared with values in *Slc39a8(+/+)* and *Slc39a8(+/neo)*. In this and subsequent figures, *bars* and *brackets* denote means ± S.E.M. *Brackets* denoting S.E.M. are present in **Fig. 1A** but are mostly covered up by the size of the *symbols*. (**B**) Photographs of (genotyped) *Slc39a8(+/+)* and *Slc39a8(neo/neo)* pups at GD11.5 (*upper left panel*), GD13.5 (*upper right*), GD16.5 (*middle both left & right*), and at PND1 (*bottom panel*). The GD16.5 fetuses (*middle left*) represent a single litter of 13 pups; the two *asterisks* in *middle left panel* denote *Slc39a8(neo/neo)* homozygotes; the other 11 were *Slc39a8(+/+)* wild-type and *Slc39a8(+/neo)* heterozygotes.

Crossing *Slc39a8(+/neo)*×*Slc39a8(+/neo)* mice resulted in healthy and viable *Slc39a8(+/+)* homozygotes and *Slc39a8(+/neo)* heterozygotes; however, poor weight gain in utero was seen in *Slc39a8(neo/neo)* homozygotes (**Fig. 1A**). The expected Mendelian ratio was observed throughout most of gestation–until GD18.5 at which time *Slc39a8(neo/neo)* homozygotes began dying, with virtually all dead by 48 h postnatally (**Table 1**). We observed striking stunted growth and pale appearance (**Fig. 1B**) in *Slc39a8(neo/neo)* homozygotes as early as GD11.5; this runted anemic appearance persisted until their demise between GD18.5 and 48 h postpartum.

**Table 1:** Mendelian ratio of offspring from GD11.5 through PND3, resulting from the *Slc39a8(+/neo)*×*Slc39a8(+/neo)* cross.

Day of life	Total viable	*(+/+)*	*(+/neo)*	*(neo/neo)*	Expected^a^ *(neo/neo)*	*P*-value
**GD11.5**	113	30	62	21	28.25	NS^a^
**GD13.5**	68	14	43	11	17.0	NS
**GD14.5**	89	22	27	29	22.25	NS
**GD16.5**	61	15	33	13	15.25	NS
**GD18.5**	108	39	59	10	27.0	<0.005
**PND1**	91	31	51	9	22.75	<0.02
**PND3**	108	32	75	1^b^	27.0	<0.0001
**Total**	638					

a **NS**, Not significant (*P*>0.05). Chi-square analysis with two degrees of freedom was used to calculate the *P*-values, assuming the genotype distribution follows the expected Mendelian ratio.

b Out of a total of 94 *Slc39a8(neo/neo)* homozygotes genotyped, one pup survived beyond 48 h; this male was runted and actually survived for more than 4 months but failed to breed; no offspring could be obtained from this single *Slc39a8(neo/neo)* survivor.

*Slc39a8(neo/neo)* newborns showed malformed craniums, hypoplastic hind limbs (forelimbs less affected), and underdeveloped eyes (**Fig. 1B**). The spleen was virtually absent. There was substantial hypoplasia of the kidneys and liver–and lungs to a lesser extent (**Table 2**). Failure of organ development was ranked as spleen most severely affected > kidney = liver > lungs smaller than normal. The *Slc39a8(neo/neo)* organ sizes (**Fig. S1**), as well as placenta, yolk sac, and total embryo (**Fig. S2**) were smaller than those of the wild-type or heterozygote. On the other hand, the size of *Slc39a8(neo/neo)* thymus, cerebrum and cerebellum was no different than wild-type (**Table 2**). The *Slc39a8(neo/neo)* heart appeared slightly larger than wild-type or heterozygotes; this increased size might reflect the severe anemia that we found in the hypomorph. Thus, in the *Slc39a8(neo/neo)*, we conclude that multiple organs fail to develop properly, from very early embryogenesis; in utero normal growth rate is also affected.

**Table 2:** Tissue/organ weights (mg/g body weight) for the three genotypes, at age GD18.5.

Genotype	Number	Liver		Lungs	Kidneys	Heart	Spleen	Thymus	Cerebrum	Cerebellum
***Slc39a8(+/+)***	28	46±3.0		19±0.97	5.4±0.29	4.4±0.52	1.5±0.53	3.1±0.62	28±2.4	15±2.4
***Slc39a8(+/neo)***	40	48±2.6		21±1.1	5.3±0.6	4.8±0.6	1.4±0.42	2.8±0.48	31±4.2	21±3.6
***Slc39a8(neo/neo)***	5	12±4.3		6.7±0.3	1.5±0.61	5.7±0.92	<0.1±0.01	2.1±0.48	35±5.0	27±11
* ***P-*** **values:**										
***(+/+)*** ** vs ** ***(neo/neo)***		<0.001		<0.001	<0.001	0.414	<0.001	0.159	0.074	0.092
***(+/neo)*** ** vs ** ***(neo/neo)***		<0.001		<0.001	<0.001	0.061	<0.001	0.181	0.201	0.237

* *P*-values were calculated using one-way ANOVA followed by the post-hoc least-significant-differences (**LSD**) test; numbers of organs ranged from 5 to 40 per group. Of all the organs surveyed, no differences were found between *Slc39a8(+/+)* pups and *Slc39a8(+/neo)* pups. In *Slc39a8(neo/neo)* pups, the weights of liver, lung, kidney and spleen were significantly lower than those from *Slc39a8(+/+)* and *Slc39a8(+/neo)* pups.

### Histology of Tissues among the Three Genotypes

Fetal yolk sac and liver showed histological differences in hematopoietic islands, as described later. GD18.5 and PND1 lung, kidney, heart, and other tissues listed in **Table 2** and **Fig. S2** showed no obvious histological differences among the three genotypes (data not illustrated), other than organ size.

### ZIP8 mRNA and Protein Levels

**Figure 2:**
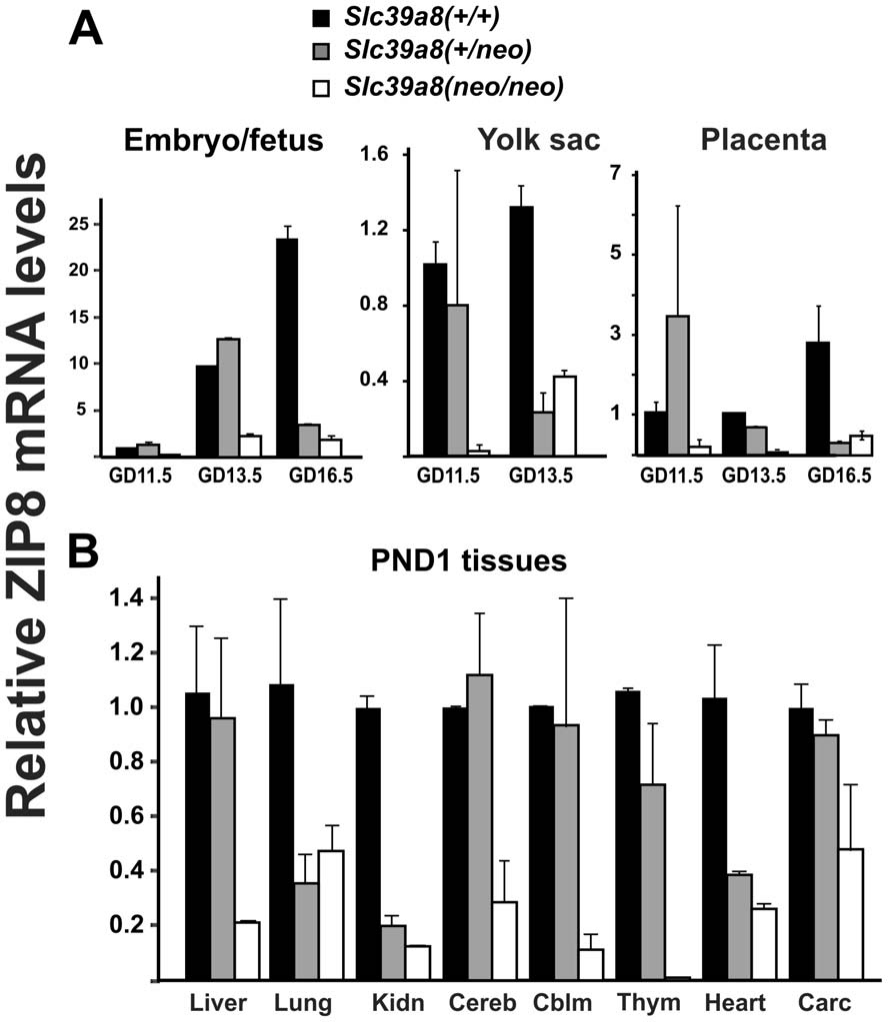
Relative ZIP8 mRNA levels in *Slc39a8(* *+* ***/*** *+* ***)*** **,** ***Slc39a8(*** *+* ***/neo)*** **and** ***Slc39a8(neo/neo)*** **genotypes.** (**A**) At three gestational ages, mRNA levels were determined in whole embryo/fetus, yolk sac, and placenta. (**B**) The mRNA was examined in PND1 liver, lung, kidney (**Kidn**), cerebrum (**Cereb**), cerebellum (**Cblm**), thymus (**Thym**), heart, and carcass (**Carc**). (“Carcass” is what remains after removal of liver, lungs, kidneys, heart, head and GI system.) Samples between five and 12 individual mice were examined. Mouse glyceraldehyde-3-phosphate dehydrogenase (**GAPDH**) was employed as the normalization control. The *Slc39a8(+/+)* wild-type ZIP8 mRNA/GAPDH mRNA ratio (left-most in each panel) and mRNA expression levels were expressed as linear fold-changes–relative to the normalized wild-type control. To lessen clutter in this figure or figure legend, all statistics are detailed in **Supplementary Data S1** online.

In the whole embryo/fetus at GD11.5, GD13.5 and GD16.5 (**Fig. 2**, *upper left*), ZIP8 mRNA levels in *Slc39a8(neo/neo)* homozygotes were significantly lower than that in the wild-type. Primers used for qRT-PCR are listed in **Table S1**. Interestingly, in some cases, ZIP8 mRNA in *Slc39a8(+/neo)* heterozygotes was significantly less than that in *Slc39a8(+/+)* wild-type mice. These observations were also seen in yolk sac at GD13.5 and placenta at GD13.5 and GD16.5 (**Fig. 2**, *upper middle & right*).

**Figure 3:**
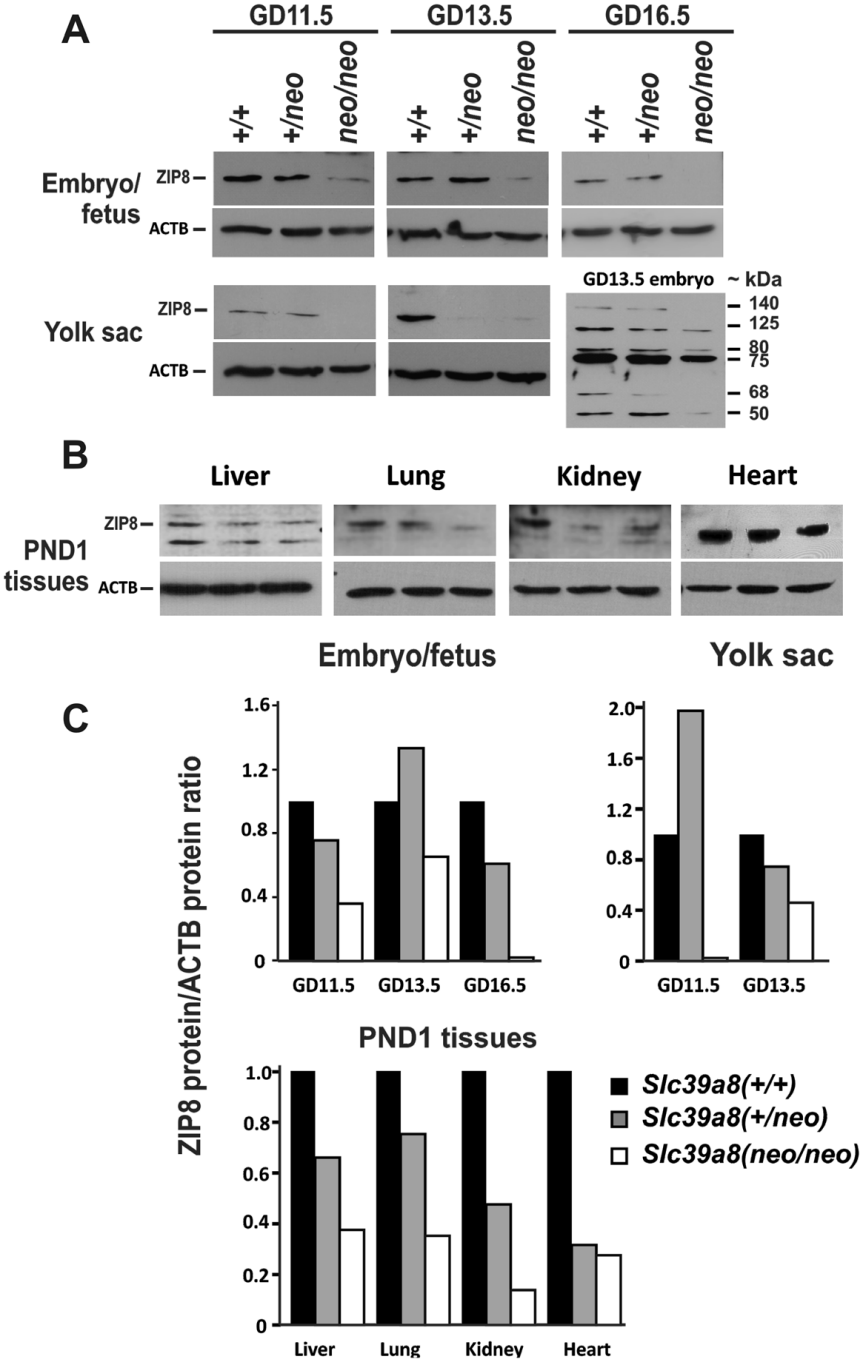
Western immunoblot analysis of ZIP8 protein (50.082 kDa) in the same tissues described in Fig. 2 . (**A**) Whole embryo/fetus and yolk sac; (**B**) Four tissues at PND1. Antibody to β-actin protein (**ACTB**; 41.7 kDa) was used as the lane-loading control. To maximize contrast, exposure times ranged from 1 min to 6 h. (**C**) Densitometric semi-quantification of Western immunoblots: whole embryo/fetus, yolk sac and four tissues at PND1. The *Slc39a8(+/+)* wild-type ZIP8 protein/ACTB protein ratio (left-most in each panel) and protein expression levels were expressed as linear fold-changes–relative to the normalized wild-type control.

In PND1 tissues (**Fig. 2**, *bottom*), ZIP8 mRNA was strikingly decreased in all tissues of *Slc39a8(neo/neo)* that were examined. The mRNA differences (**Fig. 2**) were confirmed at the protein level by Western immunoblots in whole embryo/fetus, yolk sac, liver, lung, kidney and heart (**Fig. 3**). ZIP8 protein levels appeared to follow mRNA levels quite closely in all samples; densitometric readings (**Fig. 3C**) further confirmed the Western blot data.

Note that sometimes ZIP8 mRNA and protein levels were similar in the *Slc39a8(+/+)* and *Slc39a8(+/neo)* samples, while being much less in the *Slc39a8(neo/neo)*; in other instances, ZIP8 mRNA and protein were similarly diminished in the *Slc39a8(+/neo)* and *Slc39a8(neo/neo)* samples, while being much higher in the *Slc39a8(+/+)* wild-type (**Figs. 2** **&** **3**). Most likely, this phenomenon represents ***transvection*** in the heterozygote–an epigenetic phenomenon of activation or repression that results from interaction between an allele (usually recessive) on one chromosome and the corresponding allele on the homologous chromosome [15]–[19]. This can happen specifically to one cell type, and can vary even as a function of age. Although transvection is somewhat rare, it has also been demonstrated in *Slc39a4(+/–)* mice [20].

It is intriguing that low ZIP8 mRNA and protein in an organ and the effect of low ZIP8 on organogenesis do not go hand-in-hand. In other words, low ZIP activity and small organ size were seen in liver, lung and kidney; low ZIP expression yet normal organ size were observed in cerebrum, cerebellum, thymus and heart (**Table 2** **&** **Figs. 2** **&** **3**). The severe pale phenotype was seen in *Slc39a8(neo/neo)* mice but did occur occasionally in *Slc39a8(+/neo)* mice. This observation might reflect the relative importance of endogenous ZIP8 function in different organs. The various compensatory mechanisms (in scenarios of ZIP8 loss) in different organs might also affect the final outcome of organ development.

Previous work from this lab [9] showed ZIP8 mRNA levels on Northern blots to be higher in adult mouse lung and kidney than in liver or heart. In contrast, we did not find such higher ZIP8 mRNA or protein levels in lung and kidney of newborn mice (**Fig. 2**), which may suggest distinct roles of ZIP8 in developing tissues and adult tissues.

As well as the band at the expected molecular weight for ZIP8 protein on Western immunoblots (50.082 kDa), additional heavier bands were ofttimes seen (**Fig. 3A & B**), depending on the tissue–most likely representing products of posttranslationally-modified ZIP8 protein, **e.g.** one or several events of glycosylation [10]. However, the intensity of these additional bands always appeared to correlate across genotypes with the intensity of the 50-kDa band (representing the non-glycosylated protein). To reduce clutter, we have omitted most of the higher-MW bands in **Fig. 3**. The highest number of observed bands (six), showing a direct correlation of the band intensities with genotype, is best illustrated in GD13.5 embryo (**Fig. 3A**, *lower right*). Ample evidence exists for multiple glycosylations of at least four ZIP proteins: ZIP4 [**10**]; [**21**], ZIP5 [21]; [22], ZIP8 [10], and ZIP14 [23].

### ZIP8 mRNA Length between the Two Alleles

Are there splicing differences in ZIP8 mRNA between the two alleles? We developed a PCR-based strategy to examine the entire *Slc39a8* transcript; we devised appropriate primers (**Table S2**) in order to divide the cDNA (corresponding to the mRNA) into 22 overlapping PCR fragments (**Fig. S3**). No differences in PCR fragment size were seen among the *Slc39a8(+/+)*, *Slc39a8(+/neo)* and *Slc39a8(neo/neo)* genotypes. We conclude that the ZIP8 mRNA length stays the same in the two alleles and among three genotypes.

### ZIP8 mRNA Levels in Mouse Fetal Fibroblast (MFF) and Fetal Liver Cultures

**Figure 4:**
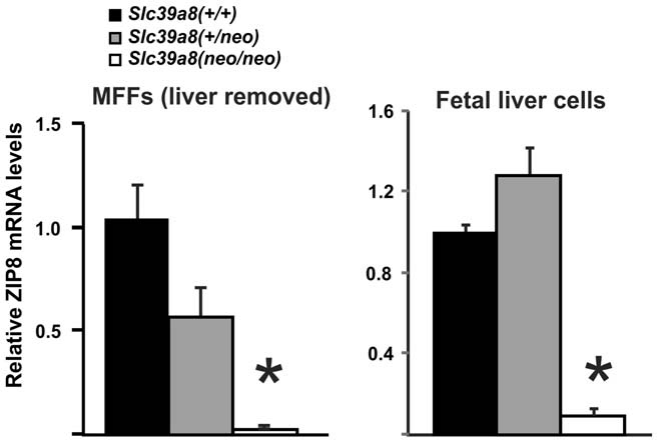
Relative ZIP8 mRNA levels in untreated *Slc39a8(* *+* ***/*** *+* ***)*** **,** ***Slc39a8(*** *+* ***/neo)*** **and** ***Slc39a8(neo/neo)*** **MFF and fetal liver cultures.** As in **Fig. 2**, the *Slc39a8(+/+)* wild-type ZIP8 mRNA/GAPDH mRNA ratio (left-most in each panel) and mRNA expression levels were expressed as linear fold-changes–relative to the normalized wild-type control. The MFFs were prepared from GD14.5 fetuses whose livers had been removed. Livers were trypsinized and cultured until they approached ∼80% confluency (<2 weeks). *P<0.0001, compared with *(+/+)* or *(+/neo)* genotypes. These data represent duplicate results from three independent experiments.

Comparing MFF cultures (**Fig. 4**, *left*) and fetal liver cultures (**Fig. 4**, *right*), ZIP8 mRNA was significantly diminished in *Slc39a8(neo/neo)* cells. These data demonstrate that MFF and fetal liver-derived cell cultures represent a faithful in vitro system for studying ZIP8 function.

### Parameters of Hematopoiesis

**Figure 5:**
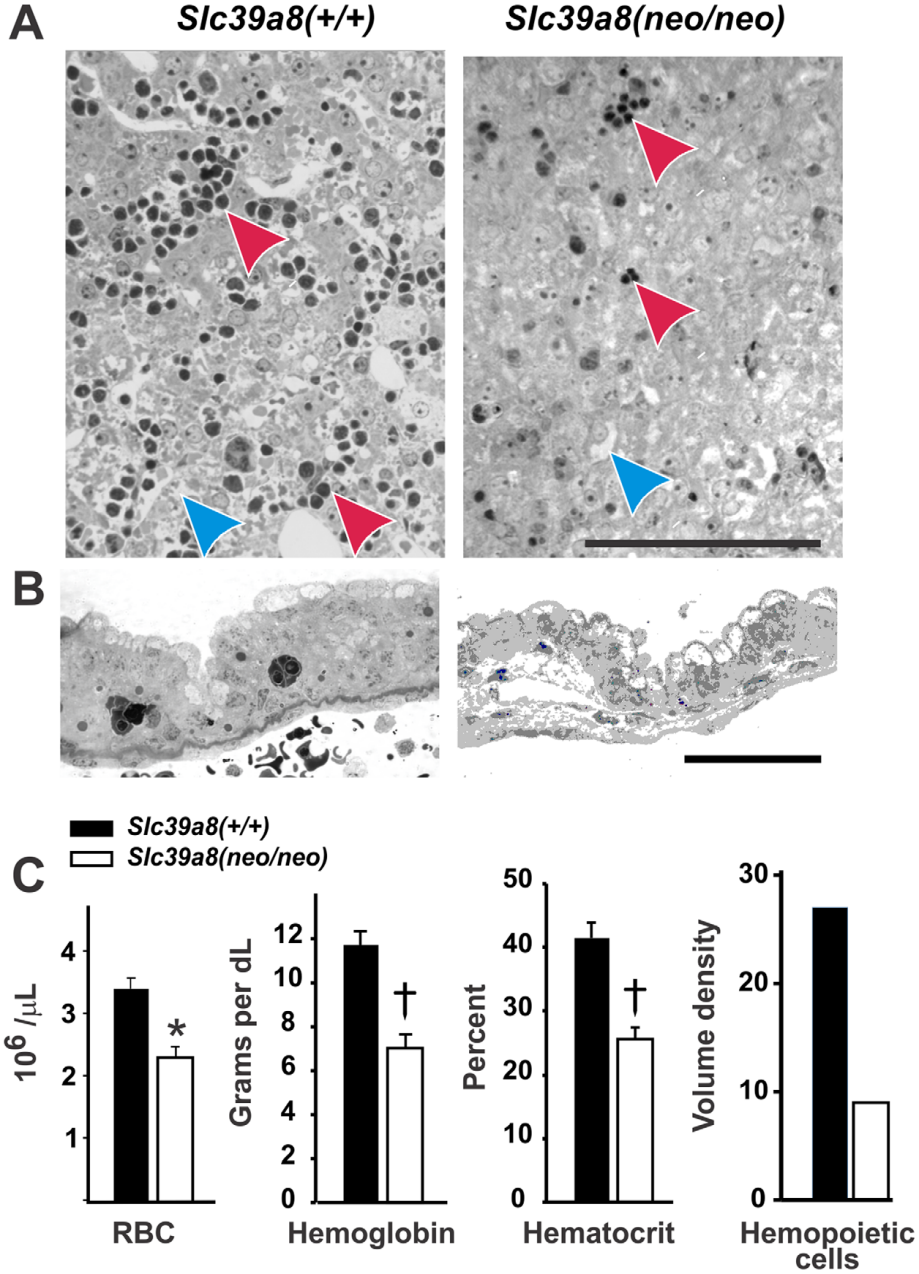
Hematopoiesis parameters. (**A**) Histology of GD16.5 liver from *Slc39a8(+/+)* vs *Slc39a8(neo/neo)* pups at GD16.5; toluidine blue O stain was used. *Red arrows* denote hematopoietic islands; *blue arrows* glycogen. Magnification = 100X. *Bar* = 100 microns. (**B**) Histology of GD13.5 yolk sac. *Bar* = 50 microns. (**C**) Histogram of red cell count (RBC; 10^6^ per µL), hemoglobin (g/dL), and hematocrit (percent) in PND1 mice. N = 8 for *Slc39a8(+/+)*, N = 13 for *(+/neo)*, and N = 7 for *(neo/neo)*. Student’s t-test was used: * *P* = 0.016. **^†^** *P* = 0.008. At *far right*, volume density (**V_d_**) of hematopoietic (**Hem**) islands/total cell number was measured in GD16.5 liver. N = 3 for *Slc39a8(+/+)*, N = 7 for *(+/neo)*, and N = 3 for *(neo/neo)*. These V_d_ differences did not reach statistical significance (*P*<0.05) between *Slc39a8(+/+)* vs *Slc39a8(neo/neo)* pups.

During mouse fetogenesis and throughout the neonatal period (**Fig. S4**), hematopoietic functions transition from the yolk sac and aorta-gonad-mesonephros region (between GD8 and GD13) to liver (GD11 to GD20), and then to spleen (after GD15.5) and marrow (after GD17.5) [24]; [25]. Mouse placenta is also a hematopoietic organ [26]. Compared with that in *Slc39a8(+/+)*, we found major decreases in the size and number of hematopoietic islands in *Slc39a8(neo/neo)* GD16.5 liver (**Fig. 5A**) and GD13.5 embryonic yolk sac (**Fig. 5B**).

These observations are consistent with the pale color seen in *Slc39a8(neo/neo)* homozygotes but not *Slc39a8(+/neo)* heterozygotes or *Slc39a8(+/+)* wild-type (**Fig. 1B**). Compared with that in *Slc39a8(+/+)* wild-type–the red blood cell count, hemoglobin levels and hematocrit were all statistically significantly decreased in *Slc39a8(neo/neo)* PND1 pups (**Fig. 5C**). The volume density of hematopoietic cells and islands (expressed as percentage of total cell number) in GD16.5 liver was more than 3-fold lower in *Slc39a8(neo/neo)* than *Slc39a8(+/+)* pups (**Fig. 5C**), another observation consistent with the anemia. These data strongly implicate an association of the strikingly decreased ZIP8 mRNA and protein levels with a severe anemia in utero and in neonates.

Comparing *Slc39a8(+/+)* and *Slc39a8(+/neo)* with *Slc39a8(neo/neo)* fetuses (**Table 3** **&** **Fig. S5**), we found that blood smears of the hypomorph showed: more erythroblasts; greater numbers of nucleated red cells (including binucleated and cells with micronuclei); hyper-condensation of the nuclei in erythroid precursors; mature neutrophils; and a suggestive premature maturation of both erythroid and myeloid precursors. Counting 1,000 cells for each animal, we found nucleated red cells + erythroblasts in whole blood averaged far fewer in *Slc39a8(+)*-containing animals than in *Slc39a8(neo/neo)* homozygotes; this dramatic increase in *Slc39a8(neo/neo)* nucleated red cells and erythroblasts persisted from GD16.5 through PND1 (**Table 3**). The percent erythroid-to-leukocyte ratio in *Slc39a8(neo/neo)* was correspondingly decreased. The number of megakaryocytes was similar in fetal and newborn liver of all three genotypes. These data indicate that smears of peripheral blood of the *Slc39a8(neo/neo)* hypomorph are abnormal, consistent with anemia.

**Table 3:** Fetal (GD16.5–18.5) and neonatal (PND1) relative and differential cell counts from peripheral blood smears*.

Nucleated RBCs + erythroblasts, as a percent of total red cells							
GD16.5		GD17.5		GD18.5		PND1	
*Slc39a8(+)*	*(neo/neo)*	*Slc39a8(+)*	*(neo/neo)*	*Slc39a8(+)*	*(neo/neo)*	*Slc39a8(+)*	*(neo/neo)*
30±0.82	44	0.39±0.17	6.0±2.8	0.49±0.11	51	<0.01	18
N = 4	1	6	5	7	1	1	1
		*P*<0.0001					


% Erythroid-to-leukocyte (myeloid + lymphoid) differential count = total erythroid×100/(total leukocyte + erythroid)							
GD16.5		GD17.5		GD18.5		PND1	
*Slc39a8(+)*	*(neo/neo)*	*Slc39a8(+)*	*(neo/neo)*	*Slc39a8(+)*	*(neo/neo)*	*Slc39a8(+)*	*(neo/neo)*
71±3.5	74	46±16	90	61±12	83	<1.0	>99
N = 4	1	6	1	8	1	1	1

* Incidence of nucleated RBCs and erythroblastic red cells to the greater RBC population was obtained from peripheral blood smears: ∼500 RBCs were counted for each animal. A differential count was also performed to assess white cells, but yielded too few white cells to produce statistically significant differences in myeloid and lymphoid cell populations. Percent of nucleated erythroid cells (red cells with nuclei in late stages of development, plus erythroblasts), compared with all leukocytes (large, medium, and small lymphocytes, monocytes, eosinophils, basophils, neutrophils, and myeloid precursors), was determined. No plasma cells and few Howell-Jolly bodies were seen. Stain used was Wright-Giemsa, prefixed with methanol.

### Analysis of Hematopoietic Cells in Liver and Yolk Sac by Flow Cytometry

**Figure 6:**
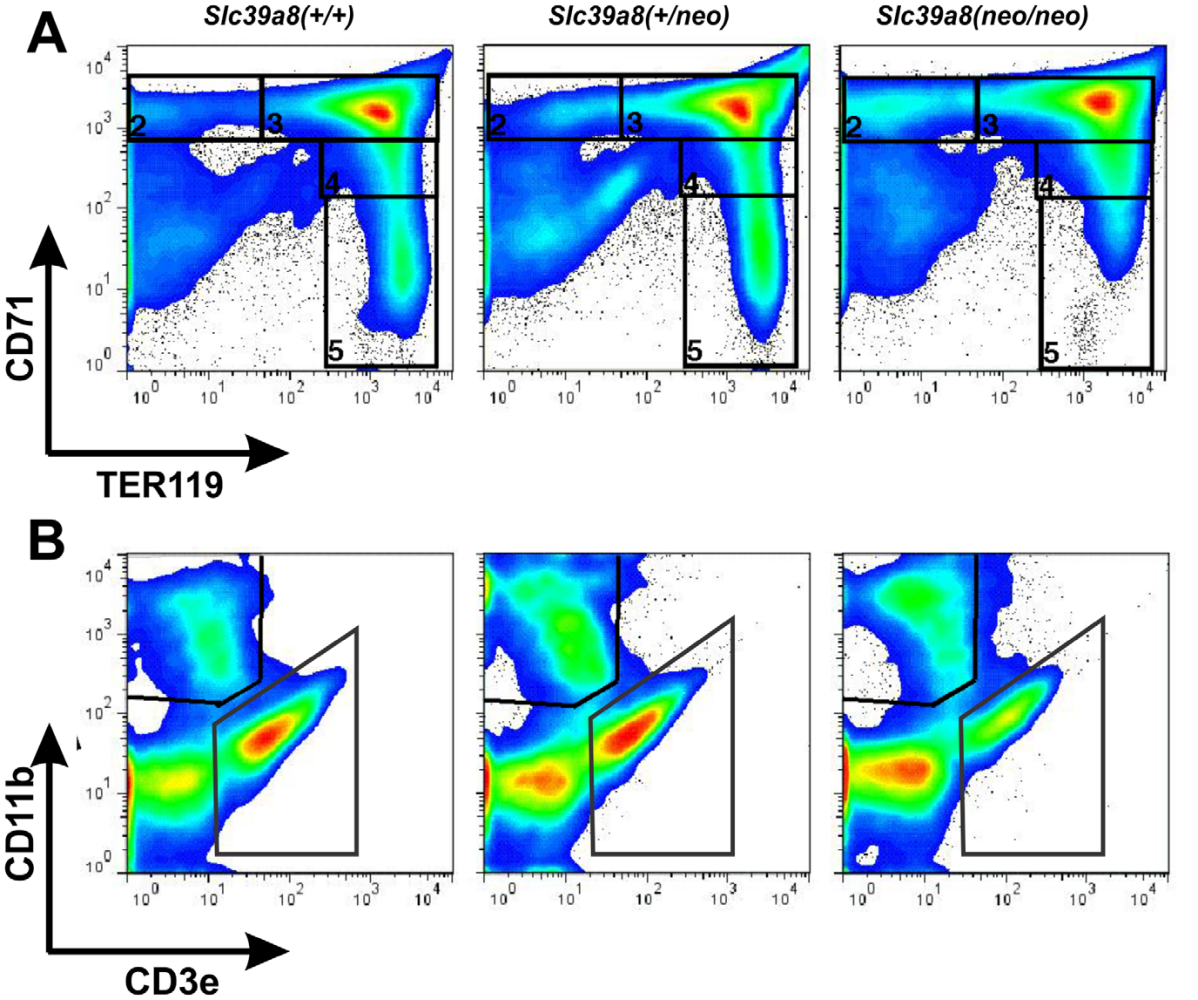
Flow cytometry. Expression of erythroid, myeloid and lymphoid markers in GD16.5 liver. These data represent typical findings from an individual animal of each genotype. (**A**) Classification and comparison of erythroid developmental stages using CD71 (transferrin receptor) and TER119 (erythroid series). Note Regions 2, 3, 4 & 5 are delineated. (**B**) Flow-cytometry analysis of the expression of CD3e (lymphoid T-cells) and CD11b (myeloid series).

To further elucidate the microscopic observations, flow-cytometry analysis of the expression of erythroid (**TER119**), transferrin receptor (**CD71**), myeloid (**CD11b**), and lymphoid (T-cells) (**CD3e**) markers on GD16.5 liver cells was carried out (**Fig. 6** **, Fig. S6;** **Tables 4** **&** **5**). Consistent with the pale appearance of *Slc39a8(neo/neo)* offspring (**Fig. 1A**), *Slc39a8(neo/neo)* livers showed significantly fewer numbers (*P* = 0.005) of total TER119-positive erythroid cells (**Table 4** **&** **Fig. S6A**). Among TER119+ cells, CD71 expression was different in *Slc39a8(neo/neo)* livers, compared with that in *Slc39a8(+/+)* or *Slc39a8(+/neo)* livers. This observation, combined with the results (described above) from the blood smears, convinced us to characterize carefully the number of cells in each stage of erythrogenesis.

**Table 4:** Frequency of erythroid, myeloid and lymphoid lineages in GD16.5 liver of the three genotypes.

Genotype	N	TER119^+^	TER119^–^/CD3e^+^	TER119^–^/CD11b^+^
***Slc39a8(+/+)***	10	85.3±0.5	4.1±0.2	3.0±0.1
***Slc39a8(+/neo)***	14	85.1±0.4	3.8±0.2	3.3±0.1
***Slc39a8(neo/neo)***	3	81.1±1.0	3.3±0.7	4.0±0.5

TER119 = erythroid; CD3e = lymphoid (T-cells); and CD11b = myeloid. Percentages of CD11b^+^ and CD3e^+^ cells were calculated as the percentage of total cells. If one adds those percentages of each row, this approaches 100% but does not completely reach 100%, because we are unable to include all of the markers necessary to identify every cell type present in the samples, due to technical limitations.

**Table 5:** Determination of erythroid developmental status, using TER119 and CD71 markers, in cells isolated from GD16.5 liver of the three genotypes.

Genotype	N	%R2	%R3	%R4	%R5
***Slc39a8(+/+)***	10	3.2±0.2	61.3±1.1	8.2±0.7	11.6±0.7
***Slc39a8(+/neo)***	14	3.4±0.1	61.0±1.4	8.1±0.4	11.9±1.0
***Slc39a8(neo/neo)***	3	6.7±0.3	64.8±1.4	8.6±0.6	3.8±0.3

Percent of cells were grouped into regions **R2** to **R5**, as shown in **Fig. 6**. Region 2 = proerythroblasts; region 3 = basophilic erythroblasts; region 4 = polychromatophilic erythroblasts; and region 5 = orthochromatophilic erythroblasts. TER119 = erythroid; CD71 = transferrin receptor. Classification of cells into R2 through R5 was based on previous studies [48]; [49], and the gates were applied to the total cells, without first gating on the Ter119^+^ population. There is a small fraction of events that do not fall into any of the gates R2 through R5, which do, however, meet the conditions for classification as TER119^+^.

Interestingly, the stage of maturity of the erythroid precursors in *Slc39a8(neo/neo)* was different from that in *Slc39a8(+/+)* and *Slc39a8(+/neo)*. Cells in different stages of erythrogenesis were discriminated, based on co-staining of CD71 and TER119, as previously described [30]. Listed in the order (**Fig. 6A**) from earliest to the most mature (**Fig. 6A**), these precursors include: early proerythroblast (Ter119^med^CD71^high^) in **Region 2**, basophilic-polychromatophilic erythroblast (Ter119^high^CD71^high^) in **Region 3**; late basophilic and polychromatophilic erythroblasts (Ter119^high^CD71^med^) in **Region 4**, and orthochromatophilic erythroblast (Ter119^high^CD71^low^) in **Region 5**. *Slc39a8(neo/neo)* showed a significantly higher proportion of cells in R2 (proerythroblasts) (*P*<0.001), but a lower proportion of cells in R5 (orthochromatophilic erythroblast) (*P*<0.001), compared with *Slc39a8(+/+)* and *Slc39a8(+/neo)*.

No striking differences among the three genotypes were found for the myeloid and lymphoid lineages. The CD3e-positive population was not gated, when plotted against CD11b (**Fig. 6B**); instead, the CD3e-positive population was gated when plotting CD3e against forward scatter to ensure that the cell size was consistent with lymphocytes. Comparing T cells by plotting them vs forward scatter or vs CD11b, the difference was insignificant. The CD11b-positive gate (**Fig. 6B**) shows a small, but significant, increase in the proportion of CD11b-positive cells among the *Slc39a8(neo/neo)* samples, suggesting that ZIP8 deficiency might affect myelogenesis in some manner. There could be an alternative explanation for the observed increase in CD11b^+^ cells–such as a mild inflammatory process in the liver. However, as noted elsewhere in this text, histology of liver showed no inflammation; moreover, serum ALT and AST levels (**Table S3**) were not elevated in *Slc39a8(neo/neo)* pups. However, CD3e-positive cells (representing T cells) were not different among the three genotypes.

We conclude that erythroid cells from *Slc39a8(neo/neo)* liver (**Fig. 6A**) are blocked in an early maturity stage (shown by the higher percentage of more immature cells in **Region 2**, plus the lower percentage of cells that are at more advanced differentiated stage in **Region 5**). These results match our observations in the blood smears (**Table 3** **& Fig. S5**). This erythroid developmental blockade is likely the reason for in utero anemia in the *Slc39a8(neo/neo)* hypomorph.

### Blood Chemistry Analyses

Consistent with the severe anemia and hematopoietic dysregulation described above, plasma iron levels and total iron-binding capacity (**TIBC**) were more than 40-fold decreased in *Slc39a8(neo/neo)*, compared with *Slc39a8(+/neo)* and *Slc39a8(+/+)* newborns (**Table S3**). To the contrary, total bilirubin (sum of conjugated plus non-conjugated) was very low and not different among the three genotypes–indicating an absence of liver pathology. In neonatal mice, serum transaminase levels are known to be highly variable, with ALT levels ranging between 100 and 600 and AST levels between 1,000 and 5,000 Units/L [27]. Serum ALT and AST levels (**Table S3**) were not elevated in the *Slc39a8(neo/neo)*, consistent with no evidence of hepatocellular or extrahepatic (especially muscle) cellular damage.

No differences among the three genotypes were observed for HDL, LDL and total cholesterol (**Table S3**). However, at least 16-fold lower triglyceride levels were seen in *Slc39a8(neo/neo)*, compared with *Slc39a8(+/neo)* and *Slc39a8(+/+)* newborns.

#### Divalent Cation Uptake in MFF and Fetal Liver-Derived Cultures

**Figure 7:**
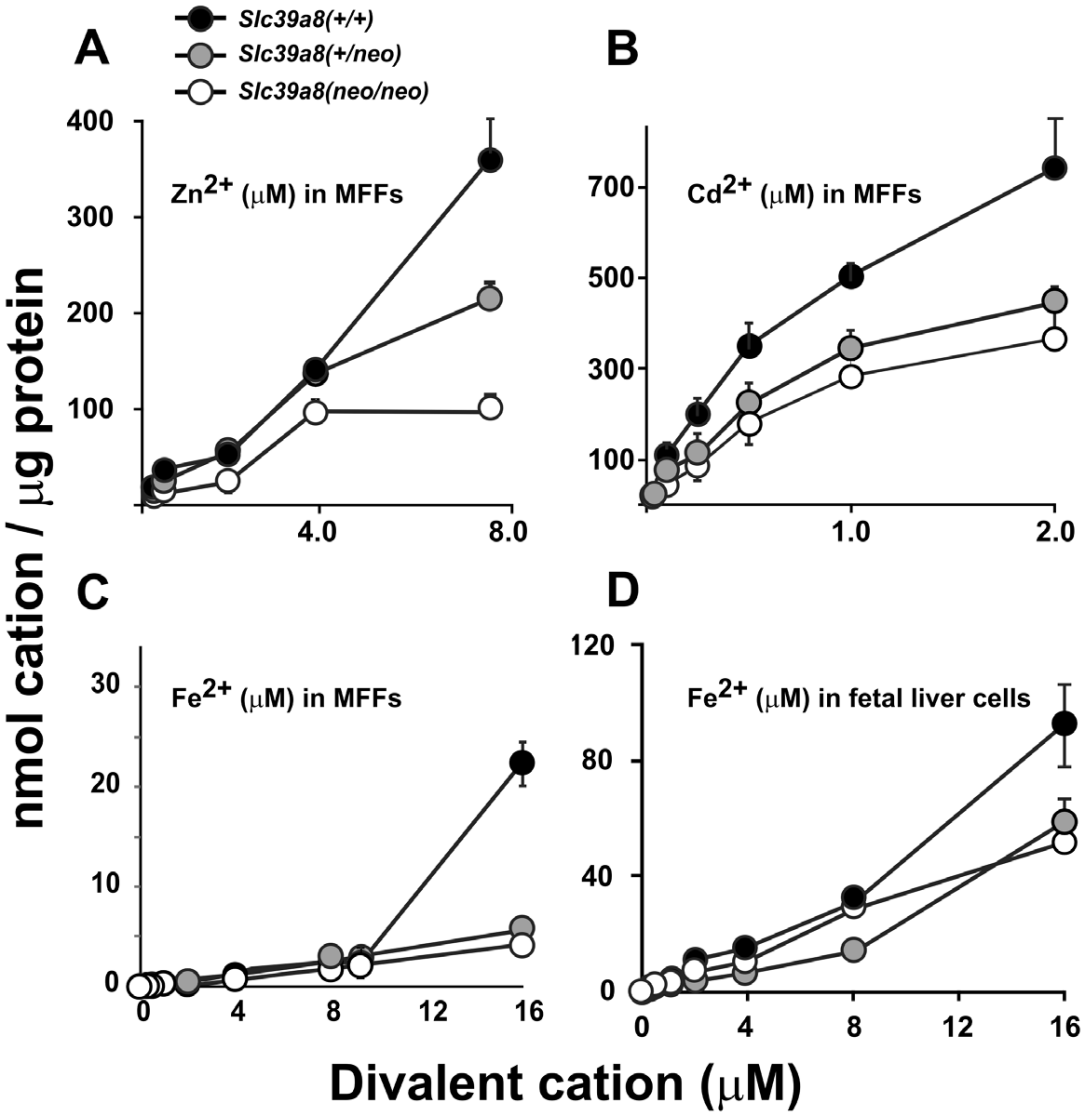
Divalent cation uptake in MFF and fetal liver cells of the three genotypes in culture. Uptake was linear for 20 min, at which time these values were recorded. These cell cultures were the same as those described in Fig. 4. At 8.0 µM **zinc in MFFs**, *(+/+)* vs *(neo/neo) P* = 0.01 and *(+/+)* vs *(+/neo) P* = 0.044. At 2.0 µM **cadmium in MFFs**, *(+/+)* vs *(neo/neo) P* = 0.002 and *(+/+)* vs *(+/neo) P* = 0.012. At 16.0 µM **iron in MFFs**, *(+/+)* vs *(neo/neo) P* = 0.003 and *(+/+)* vs *(+/neo) P* = 0.015. At 16.0 µM **iron in fetal liver cells**, *(+/+)* vs *(neo/neo) P* = 0.031.

Studying MFF cultures derived from the three genotypes, we found the *Slc39a8(neo)* allele to be associated with decreased zinc (**Fig. 7A**), cadmium (**Fig. 7B**), and iron (**Fig. 7C**) uptake. Fetal liver cells (which include hepatocytes and hematopoietic progenitor cells, as well as interstitial cells) were cultured separately from MFFs; again, we found the *Slc39a8(neo)* allele was associated with decreased iron uptake (**Fig. 7D**). As were noted for some samples with ZIP8 mRNA (**Fig. 2**) and protein (**Fig. 3**) levels, the divalent cation uptake in the *Slc39a8(+/neo)* heterozygote exhibited a gene-dose behavior in zinc-exposed MFFs (**Fig. 7A**) and was not statistically different from the *Slc39a8(neo/neo)* in the other three panels. These discrepancies might reflect the phenomenon of transvection.

#### Divalent Metal Content in PND1 Tissues

**Figure 8:**
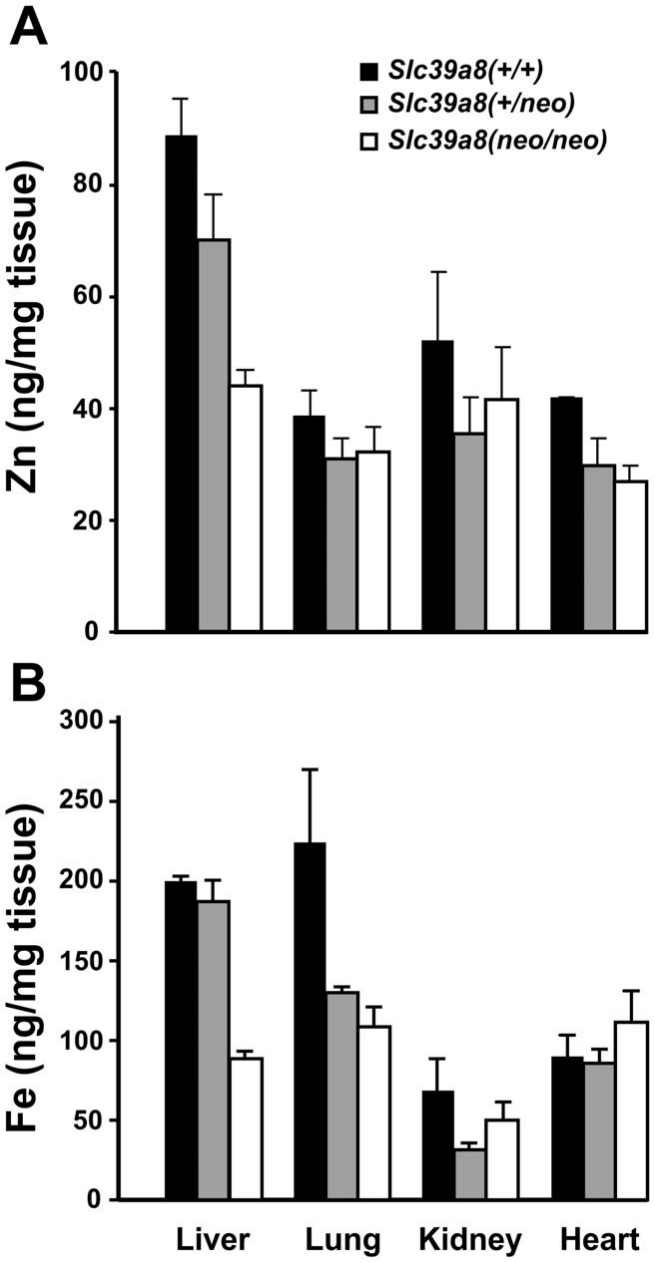
Divalent cation content (determined by ICP-MS) in four tissues at PND1. (**A**) zinc; (**B**) iron. Comparing two, we used Student’s t-test. For more than two, we used all-pairwise multiple-comparison procedures (Holm-Sidak method). To lessen clutter in the figure, all statistics are detailed in **Supplementary Data S1** online.

ICP-MS analysis (**Fig. 8A**) revealed that presence of the *Slc39a8(neo)* allele was statistically significantly associated with decreased zinc content in liver and heart, but not lung or kidney. This lack of difference for zinc in kidney is not surprising, however, because–like many other transporters in the renal proximal and distal tubules–it may take several weeks following parturition for ZIP8 to be fully expressed and fully functioning in the kidney [28].

The *Slc39a8(neo)* allele was also associated with lower iron content in liver and lung but not kidney or heart (**Fig. 8B**). Again, the *Slc39a8(+/neo)* phenotype resembled the *Slc39a8(+/+)* wild-type in some tissues, whereas the *Slc39a8(+/neo)* phenotype resembled more closely the *Slc39a8(neo/neo)* genotype in others.

We also carried out ICP-MS analysis for copper and manganese content in these same tissues, and no significant differences among the three genotypes were found: for copper, values ranged between 15.5 and 29 ng/mg tissue for liver, 2.9 and 4.2 for lung, 4.3 and 6.2 for kidney, and 4.9 and 6.1 for heart. In addition, we measured manganese content, which was undetectable (<1.0 ng/mg) in liver, lung, kidney and heart (not shown).

### Assay for 5-Aminolevulinic Acid Dehydratase (ALAD) Activity

Altered zinc transport in utero, leading to the phenotype described in the *Slc39a8(neo/neo)* hypomorph, is likely to be caused by inactivation of one or more critical zinc-dependent enzymes and/or transcription factors. One outstanding candidate might be ALAD, also called porphobilinogen synthase (**PBGS**), an enzyme requiring zinc as a cofactor; in a state of severe zinc deficiency, ALAD could thus become the rate-limiting step, resulting in a lack of hemoglobin synthesis (**Fig. S7**).

We analyzed ALAD activity in pooled PND1 livers from the three genotypes. For each set of livers, native activity (non-activated assay) was compared with zinc-activated activity; this can be expressed as the Relative Index (**R.I.**) of ALAD activity:



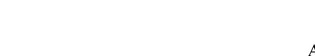
.

The R.I. should be a sensitive indicator of differences in enzyme activity due to low levels of zinc in liver: the larger the R.I., the greater the difference in ALAD activity between the zinc-activated and non-activated systems. Therefore, the larger the R.I., the greater the effect of zinc deficiency.

**Figure 9:**
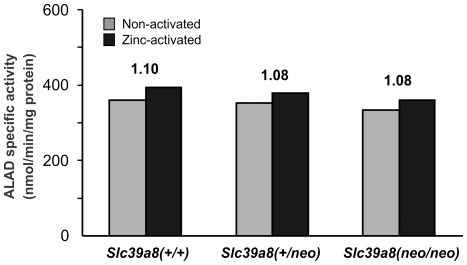
Comparison of ALAD specific activity from PND1 liver among the three genotypes for the non-activated vs zinc-activated assays. Values shown *at top* of each set of *bars* correspond to the Relative Index (ratio reflecting proportion in which the activity might increase upon zinc addition). *Brackets* denoting S.E.M. cannot be added because only two pools per genotype were assayed.

Two sets of pooled livers per genotype were incubated (**Fig. 9**); a maximal effect of zinc enhancement of ALAD was found at 15 min, compared with an incubation time of 30 min (data not shown) when enzyme kinetics approached saturation levels, and zinc activation was noticeably less. We tested differences in variously added zinc concentrations between 8 and 400 µM ZnCl_2_, and we chose to add 18 µM zinc (which is the physiologically normal plasma zinc level) for the zinc-activated assay. We found the R.I. to be greatest around 18 µM added zinc, with decreases below native activity when zinc concentrations exceeded 50–60 µM.

The mean ALAD activities (non-activated assay) ranged between 334 and 362 nmol/min/mg protein and showed no differences among the three genotypes (**Fig. 9**). Also, the R.I. values for the *Slc39a8(+/+)*, *Slc39a8(+/neo)* and *Slc39a8(neo/neo)* newborns were not significantly different (1.10, 1.08 and 1.08, respectively); our results indicate that zinc has no detectable effect. These data indicate that the zinc-dependent ALAD appears not to represent the underlying cause of the *Slc39a8(neo/neo)* dysregulation in hematopoiesis.

### Attempt to Rescue *Slc39a8(neo/neo)* Homozygotes via Breeding with BTZIP8-3 Mice

Finally, we wished to confirm that the observed phenotype (anemia and newborn lethality) was indeed due to the *neo*-cassette-mediated down-regulation of *Slc39a8* gene expression during mouse embryo development. This lab had previously created the BAC-transgenic *BTZIP8-3* mouse line, which carries three extra copies of the 129S6/SvEvTac *Slc39a8* gene in addition to its normal diploid number of C57BL/6J *Slc39a8* genes. The transgenic BAC allele (168,722 bp) appears to have all the necessary regulatory elements to ensure proper tissue and temporal expression of the *Slc39a8* gene [9]. We postulated that addition of three *Slc39a8* BAC transgenes (located elsewhere in the genome) might be able to rescue the observed phenotype during in utero and early postnatal development.

Studying the Slc39a8(+/neo)×BTZIP8-3(+/–) cross, there are six possible genotype outcomes (**Table 6**). Although the Slc39a8(+/+)_BTZIP8-3(+), Slc39a8(+/+)_BTZIP8-3(–), Slc39a8(+/neo)_BTZIP8-3(+), Slc39a8(+/neo)_BTZIP8-3(–), and Slc39a8(neo/neo)_BTZIP8-3(+) genotypes were found in the expected Mendelian ratio, there were no surviving Slc39a8(neo/neo)_BTZIP8-3(–) pups. The Slc39a8(neo/neo)_BTZIP8-3(+) pups, however, appeared normal in growth, had no anemia, and later they produced fertile offspring; in fact, crossing these pups produced healthy viable Slc39a8(+/neo) heterozygous offspring (not shown).

**Table 6:** Rescue of *Slc39a8(neo/neo)* pups by breeding *Slc39a8(+/neo)* and *BTZIP8-3* mice.

*Slc39a8*	(+/+)		(+/neo)		(neo/neo)		Total
***BTZIP8-3***	(+)	(–)	(+)	(–)	(+)	(–)	
***Surviving offspring***	4	6	7	9	4	0^a^	30

a Pups (four litters) were genotyped on PND14. Chances of finding no surviving offspring having the *Slc39a8(neo/neo)_BTZIP8-3*(–) genotype, out of a total of 30 live births, gave a *P*-value of <0.001 (chi-square analysis, with two degrees of freedom).

Hence, presence of additional *Slc39a8* genes–located elsewhere in the genome–overrides the effects of the *Slc39a8(neo/neo)* genotype. Thus, it can be concluded that the observed phenotype of stunted growth, dysregulation of hematopoiesis, anemia, multiple-organ failures in development, and in utero and neonatal lethality is indeed due to lowered ZIP8 caused by the *Slc39a8(neo/neo)* genotype.

## Discussion

Zinc plays a critical role in intracellular signal transduction [29], cell cycle and proliferation [30], processes involving development and differentiation [31], and maintaining normal function of numerous transcription factors [32]. In the human or rodent, there are almost 100 zinc-dependent enzymes [33] and more than 2,000 zinc-dependent transcription factors [34]. Because these enzymes and factors carry out important functions throughout development–often exerting cell-specific effects on morphogenesis, growth, and differentiation, the embryo’s ability to control zinc homeostasis becomes essential from the blastocyst stage onward [35]. In the present study we have described an intriguing phenotype in the *Slc39a8(neo/neo)* mouse that provides valuable insight into the importance of ZIP8-mediated zinc uptake in utero. Among many and possibly redundant zinc transporters in the adult animal, ZIP8 appears to be indispensible for embryo development; a deficient ZIP8 transporter results in failure of multiple-organ development and dysregulated hematopoiesis in utero.

The critical role of ZIP8 in hematopoiesis is the most unique aspect of the present study. There is an increase in ZIP8 expression in whole embryo between GD11.5 to GD16.5, whereas ZIP8 expression in yolk sac decreases between GD11.5 and GD13.5 (**Figs. 2** **&** **3**); this coincides with the time during which early-embryo hematopoiesis is known to transition (**Fig. S4**) from yolk sac to fetal liver [24]. Therefore, these data are consistent with an hypothesis that ZIP8 is functioning during hematopoiesis–first in yolk sac, and later as hematopoiesis in fetal liver. There is also evidence suggesting that the placenta is important in hematopoiesis [26]. Considering the pronounced pale appearance of *Slc39a8(neo/neo)* embryos or fetuses, the anemia parameters that were measured in GD16.5 fetuses and PND1 neonates (**Fig. 5**) might in fact be an under-extrapolation of the degree of anemia in the developing embryo/fetus.

Iron deficiency following ZIP8 loss could be a major reason for the anemia phenotype. Fe^2+^ is essential for heme synthesis and erythropoiesis. The current understanding is that cells take up iron via the Fe^3+^-transferrin complex: the Fe^3+^-transferrin complex can bind to specific receptors present on the cell surface, which then induce endocytosis [36]. To date, there is no divalent (Fe^2+^) transporter identified that is indispensable for iron uptake in embryonic and fetal hematopoietic organs–including yolk sac and fetal liver. Our study suggests the existence of such a pathway.

The pronounced anemia phenotype observed in *Slc39a8(neo/neo)* mice may also be an outcome of dysregulation of Zn^2+^-dependent transcriptional events in hematopoiesis. This idea is consistent with the emerging consensus that Zn^2+^ acts as a “second-messenger” in cell-cell signaling [37] including the central nervous system [38]; [39] and immune system [40]; to date, no one has published evidence in support of Zn^2+^ as a second-messenger during in utero hematopoiesis.

Thymic atrophy, lymphopenia, and compromised cell- and antibody-mediated responses that cause increased rates of infections of longer duration are the immunological hallmarks of zinc deficiency in adult humans and mice [41]; however, in *Slc39a8(neo/neo)* fetuses and newborns, we did not observe any decrease in thymus weight (**Table 2**)–suggesting that chronic zinc deficiency in adults is distinctly different from what is seen here in our mouse in utero model, with regard to effects of ZIP8 deficiency on hematopoiesis.

Whereas a visibly pale and smaller *Slc39a8(neo/neo)* homozygote is apparent from GD11.5 onward (**Fig. 1**), we found in utero lethality and resorption did not become significant until GD18.5 (**Table 1**). The precise cause of death between GD18.5 and 48 h remains unclear; however, the chronic anemia in utero, plus the serious underdevelopment of several organ systems (spleen, liver, kidney and lung) are most likely contributing factors.

Decreased total zinc and iron content occurs in many PND1 *Slc39a8(neo/neo)* tissues (**Fig. 8**). It is highly likely that decreases in zinc and iron load in the developing tissues (in utero) are more severe than those measured in PND1, because in the surviving PND1 pups, compensatory pathways that could counter the effect of ZIP8 loss may already have been selected for (*e.g.* up-regulation of other zinc transporters).

The data in **Table 6** confirm unequivocally that the *neo*-cassette-mediated dysregulation of *Slc39a8* gene expression is responsible for the observed phenotype during mouse embryo development. If additional transgenic *Slc39a8* alleles are present in the offspring [*e.g.* as occurs in *Slc39a8(neo/neo)_BTZIP8-3(+)* pups], the mice are viable and fertile. This experiment strongly argues that the lack of a functional ZIP8 is both necessary and sufficient for all the defects associated with this hypomorphic phenotype.

The finding of strikingly lowered serum triglyceride levels (**Table S3**) is intriguing, in light of a recent genome-wide association (**GWA**) study showing that a single-nucleotide polymorphism (**SNP**) in the *SLC39A8* gene–causing a missense (**p.Ala391Thr**) mutation–is associated with low HDL-cholesterol, elevated blood pressure, increased body mass index, and abnormal natriuric peptide levels; 8% of the eight populations studied carried the Thr391 mutation [42]. The role of ZIP8 in lipid metabolism therefore warrants further investigation.

In a case-control GWA study in Spain of 476 schizophrenia patients and 447 control subjects, and then further corroborated in a second sample comprising 4,069 cases and 15,128 control subjects of European origin, a different highly significant nonsynonymous SNP within the *SLC39A8* gene was described; the allelic frequency of this missense mutation is 0.34 in Europeans [43]. Moreover, a *de novo* deletion affecting *SLC39A8* and 10 additional genes was reported in a patient with nonsyndromic mental retardation {354}. These clinical findings suggest that ZIP8 might provide a critical function within the central nervous system (**CNS**). Although we found strikingly lowered ZIP8 mRNA (**Fig. 2**) and protein in cerebrum and cerebellum of the *Slc39a8(neo/neo)* hypomorph, the brain was not decreased in size (**Table 2**), but we did note a misshapen cranium. Thus, a possible role of ZIP8 in the CNS should be studied further.

To elucidate the role of ZIP8 in specific cell types, conditional *Slc39a8(–/–)* knockout lines need to be generated. Recently, using either the *Slc39a8(neo)* allele or an *Slc39a8(f)* floxed allele, we have successfully generated tissue-specific knockout lines in which ZIP8 expression can be ablated in hepatocytes or in renal proximal tubular epithelial cells (*unpublished data*); creation of alveolar epithelial-specific and neuron-specific conditional knockout lines are underway. These lines should help provide insight into the function of ZIP8 in heart disease and CNS disorders.

Finally, this study demonstrates the value of characterizing a hypomorphic allele instead of a conventional knockout mouse. ZIP8 is expressed as early as the gastrula stage [45] and in visceral endoderm at GD7.5 [46]. In fact, ZIP8 is used as a potential indicator of cell differentiation (self-renewal-related signaling) in embryonic stem cells [47]. Hence, it is very likely that a *Slc39a8(–/–)* global conventional knockout would die very early during development–making studies of the function of this gene virtually impossible–whereas the *Slc39a8(neo/neo)* pup, surviving until the neonatal period, provides us with a larger window of time for studying ZIP8 function during in utero and neonatal growth, multi-organ development, and hematopoiesis. Any of the mouse lines described in this paper are available to interested colleagues.

## Materials and Methods

### Animals

All cloning details were described previously [14]. For all in utero studies, the morning on which the vaginal plug was found is considered gestational day-0.5 (**GD0.5**). Individual *Slc39a8(+/+)*, *Slc39a8(+/neo)* and *Slc39a8(neo/neo)* whole embryos/fetuses, placentas, and yolk sacs were collected and genotyped at various time-points between GD11.5 and GD18.5; on postnatal day 1 (**PND1**), we also collected numerous tissues from genotyped pups for further analysis. All mouse experiments were conducted in accordance with the National Institutes of Health standards for the care and use of experimental animals and the University Cincinnati Medical Center Institutional Animal Care and Use Committee [protocol #11-09-12-01; approved 4 Sept 2011 → 3 Sept 2014].

### Methods and Techniques

All methods and any associated references are available in the **Supplementary Data S1** online.

## Supporting Information

Figure S1 **Comparison of size of six PND1 organs among the three genotypes**. Inserted ruler is measured in cm and mm. The *Slc39a8(neo/neo)* organ can sometimes be seen as more pale than that in the heterozygote or wild-type.
(TIF)

Figure S2 **Comparison of size of GD13.5 placenta, yolk sac and whole embryo among the three genotypes**. Again, sometimes the *Slc39a8(neo/neo)* appears more pale than the heterozygote or wild-type.
(TIF)

Figure S3 **PCR gel (2% agarose).** Twenty-two PCR fragments encompassing the entire ZIP8 mRNA, in which the sizes of all three genotypes can be compared. Because the *neo* mini-cassette is located in distal intron 3, mRNA fragments located near this region (sequences 201-006 to 201-10) might show alterations in length; however, no changes were observed there or anywhere else.
(TIF)

Figure S4 **Diagram of tissues involved during mouse embryonic and fetal hematopoiesis.** Vertical axis denotes the magnitude of contribution of each organ to hematopoiesis. **AGM**, aorta-gonad-mesonephros region [*modified from* http://commons.wikimedia.org/wiki/User:Dietzel65].
(TIF)

Figure S5 **Representative blood cells from GD16.5 and GD17.5 fetuses of all three genotypes.** Individual cells were cut and pasted into a montage for each animal: *top row*, erythroid precursors; *2nd*, nucleated erythroid precursors; *3rd*, red cells that have ejected their nuclei; *bottom row*, myeloid precursors. The contrast, hue, saturation and brightness were adjusted in Corel Draw. *Arrows* point to a binucleated red cell (*left*) and a micronucleus (*right*). *Bar* (*lower middle panel*) denotes 5 microns.
(TIF)

Figure S6 **Flow cytometry.** Expression of erythroid, myeloid and lymphoid markers in GD16.5 liver from the same single individual from each of the three genotypes, as evaluated in **Fig. 6**. (**A**) Number of cells that are positive for the TER119 marker. (**B**) Number of cells that are positive for the CD71 marker. Numbers of TER119+ and CD71+ cells in *Slc39a8(neo/neo)* were significantly (*P*<0.05) lower than those in the *Slc39a8(+/+)* and *Slc39a8(+/neo)* genotypes.
(TIF)

Figure S7 **Illustration of the hemoglobin biosynthetic pathway, showing feedback repression of ALAS by heme.** (Heme ultimately binds with one of several forms of globin to make hemoglobin.) **Succinyl Co-A**, combination of succinic acid and coenzyme-A. **ALAS**, 5-aminolevulinic acid synthase. **ALAD**, 5-aminolevulinic acid dehydratase. **PBGS**, porphobilinogen synthase (trivial name). **HMB**, hydroxymethylbilane. **URO**, uroporphyrinogen. **COPRO**, coproporphyrinogen. **PROTO**, protoporphyrinogen.
(TIF)

Table S1 Primers used for qRT-PCR.
(DOC)

Table S2 Primers used to test ZIP8 mRNA (transcript) fragment sizes between the *Slc39a8(+)* and *Slc39a8(neo)* alleles.
(DOC)

Table S3 Comparison of blood chemistry tests among the three genotypes at age of PND1.
(DOC)

Data S1 All Materials and Methods are described in detail. In addition, P-values for all comparisons of data in **Figures 2** **&** **8** are provided. Finally, seven supplemental figures and three supplemental tables are included.
(DOC)
