# Supplementary figures and images for: ZIP8 Zinc Transporter: Indispensable Role for Both Multiple-Organ Organogenesis and Hematopoiesis In Utero
Source: PLoS One. 2012 May 1;7(5):e36055. doi: 10.1371/journal.pone.0036055 (PMC3341399; doi:10.1371/journal.pone.0036055)

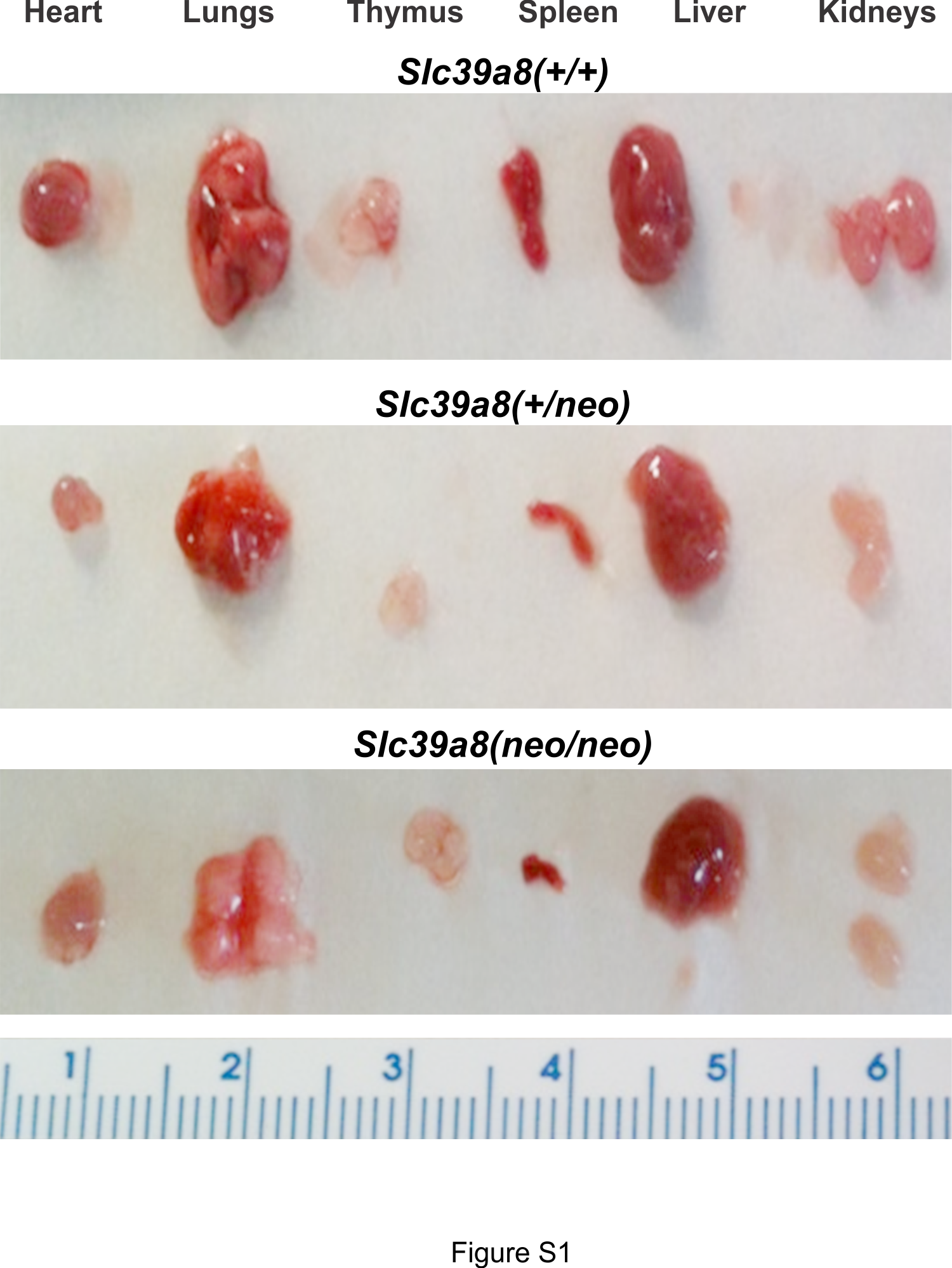

Supplement: Figure S1 — Comparison of size of six PND1 organs among the three genotypes. Inserted ruler is measured in cm and mm. The Slc39a8(neo/neo) organ can sometimes be seen as more pale than that in the heterozygote or wild-type. (TIF) [file pone.0036055.s001.tif]

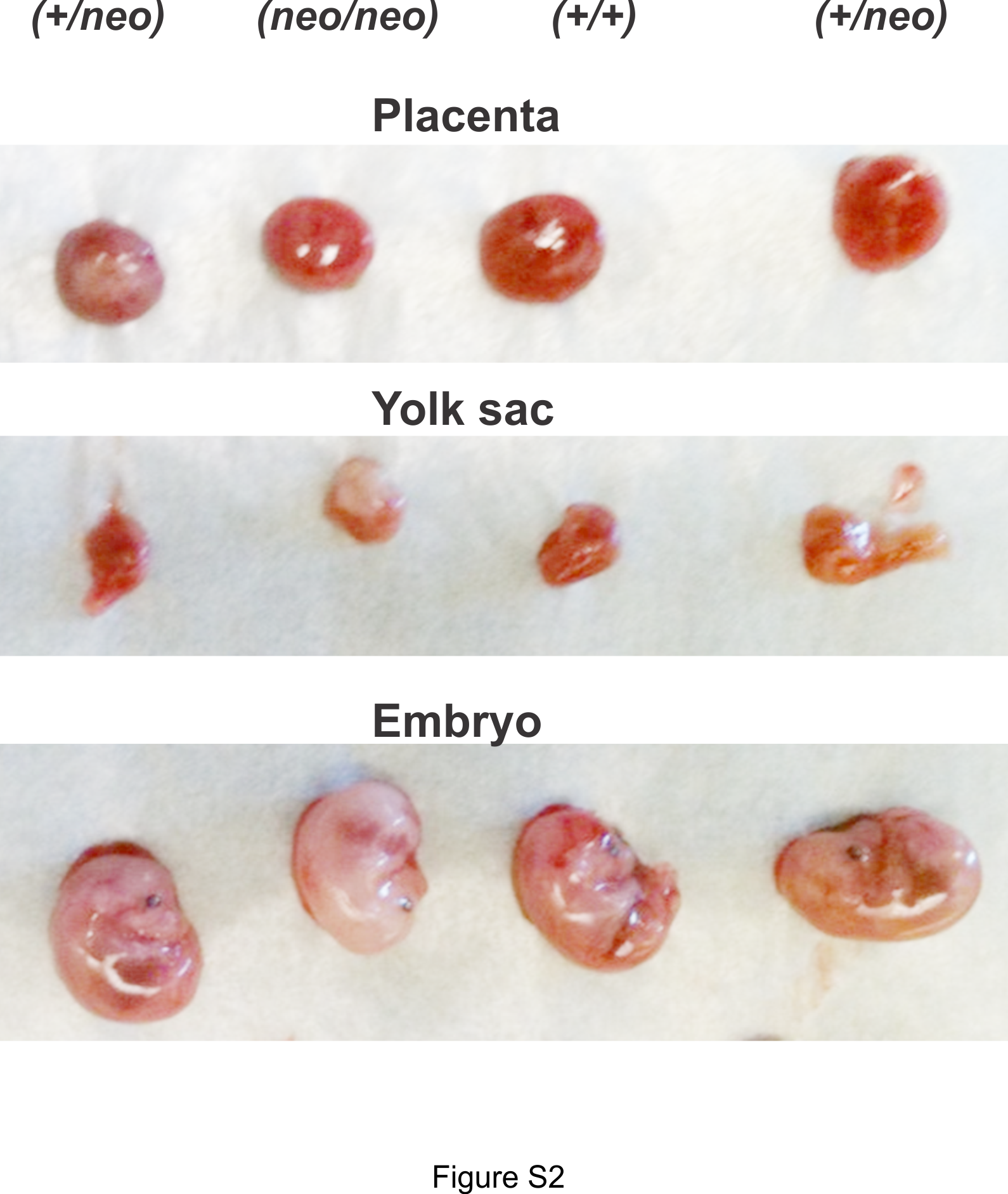

Supplement: Figure S2 — Comparison of size of GD13.5 placenta, yolk sac and whole embryo among the three genotypes. Again, sometimes the Slc39a8(neo/neo) appears more pale than the heterozygote or wild-type. (TIF) [file pone.0036055.s002.tif]

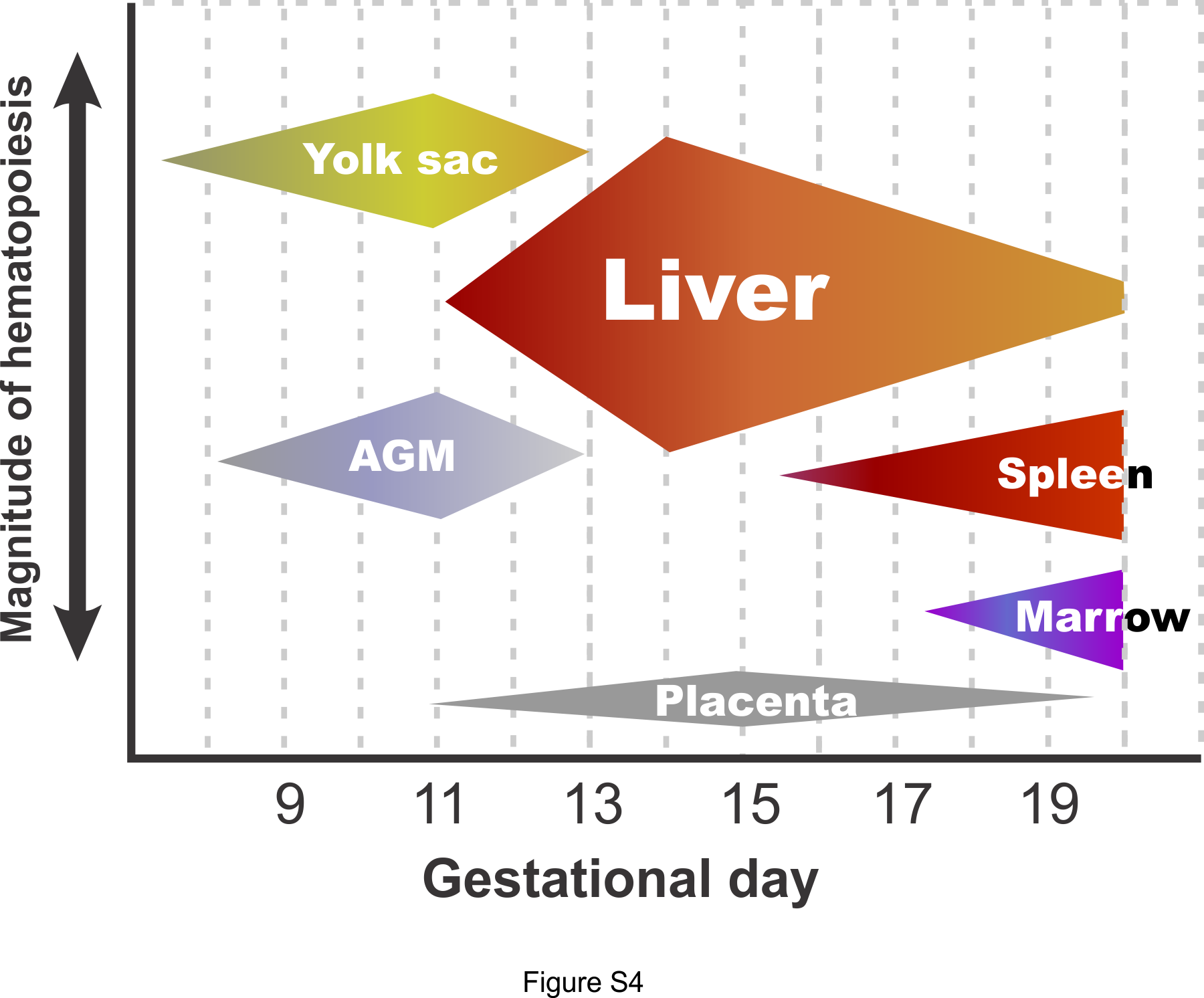

Supplement: Figure S4 — Diagram of tissues involved during mouse embryonic and fetal hematopoiesis. Vertical axis denotes the magnitude of contribution of each organ to hematopoiesis. AGM, aorta-gonad-mesonephros region [modified from http://commons.wikimedia.org/wiki/User:Dietzel65]. (TIF) [file pone.0036055.s004.tif]

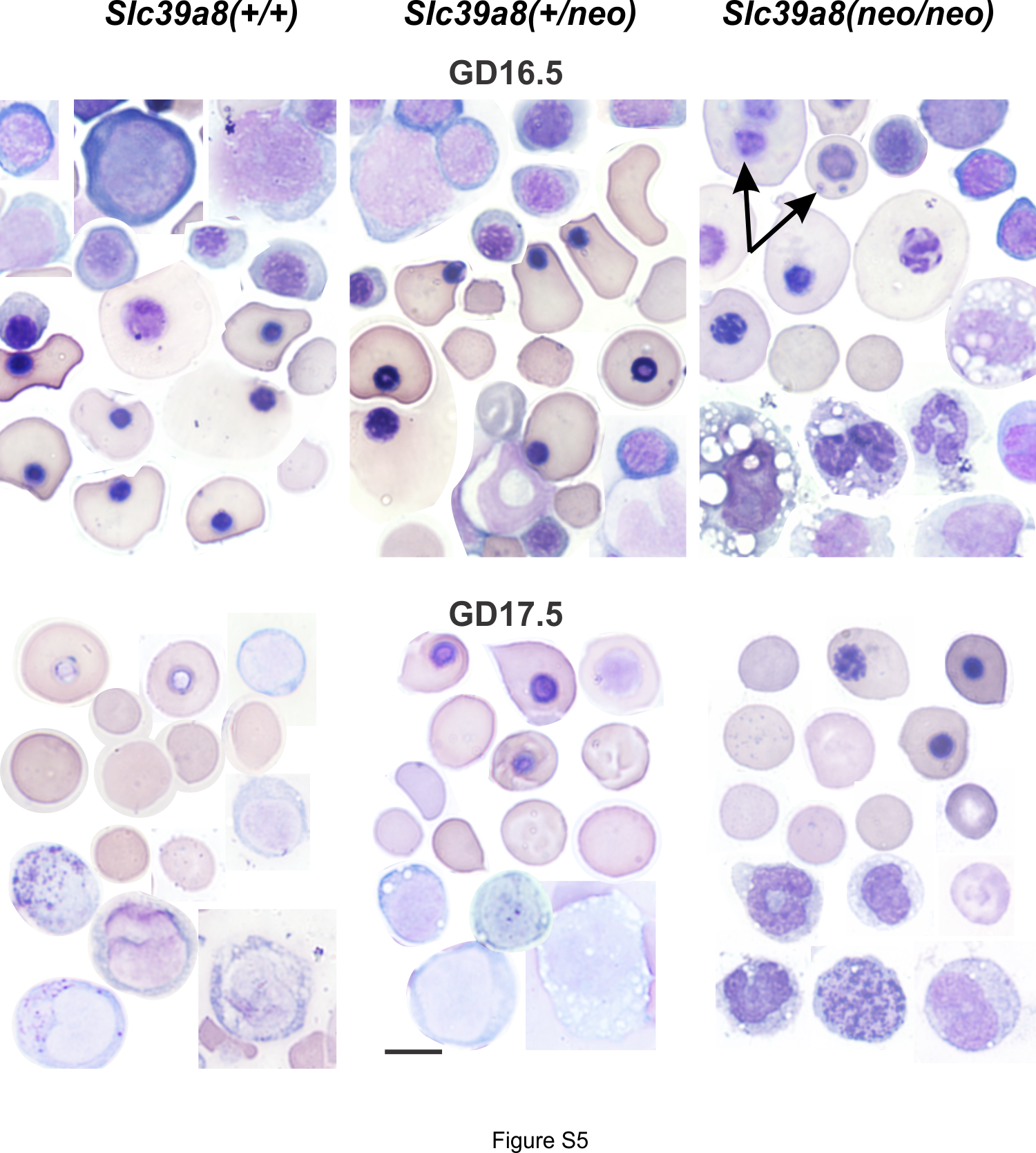

Supplement: Figure S5 — Representative blood cells from GD16.5 and GD17.5 fetuses of all three genotypes. Individual cells were cut and pasted into a montage for each animal: top row, erythroid precursors; 2nd, nucleated erythroid precursors; 3rd, red cells that have ejected their nuclei; bottom row, myeloid precursors. The contrast, hue, saturation and brightness were adjusted in Corel Draw. Arrows point to a binucleated red cell (left) and a micronucleus (right). Bar (lower middle panel) denotes 5 microns. (TIF) [file pone.0036055.s005.tif]

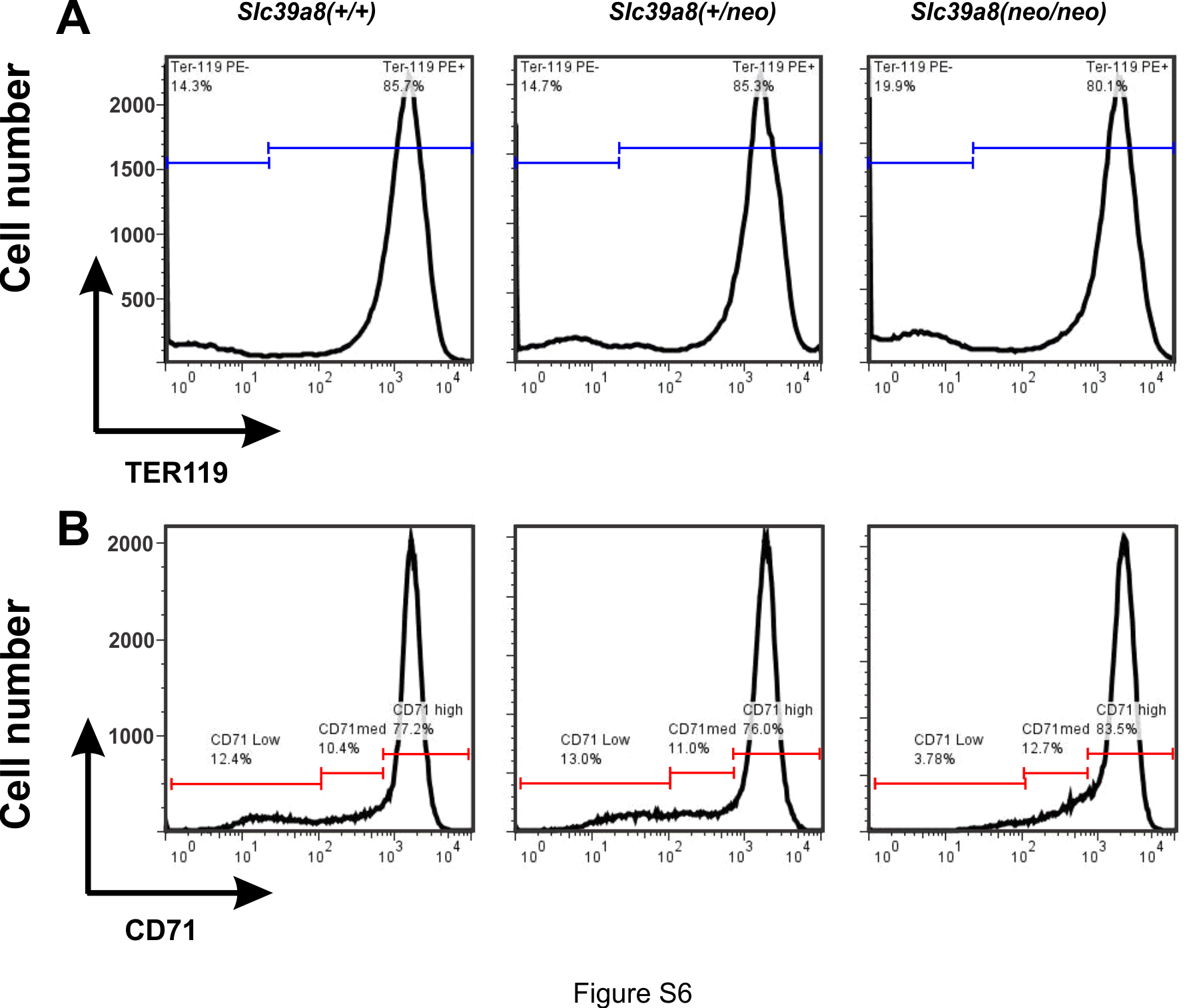

Supplement: Figure S6 — Flow cytometry. Expression of erythroid, myeloid and lymphoid markers in GD16.5 liver from the same single individual from each of the three genotypes, as evaluated in Fig. 6 . (A) Number of cells that are positive for the TER119 marker. (B) Number of cells that are positive for the CD71 marker. Numbers of TER119+ and CD71+ cells in Slc39a8(neo/neo) were significantly (P<0.05) lower than those in the Slc39a8(+/+) and Slc39a8(+/neo) genotypes. (TIF) [file pone.0036055.s006.tif]

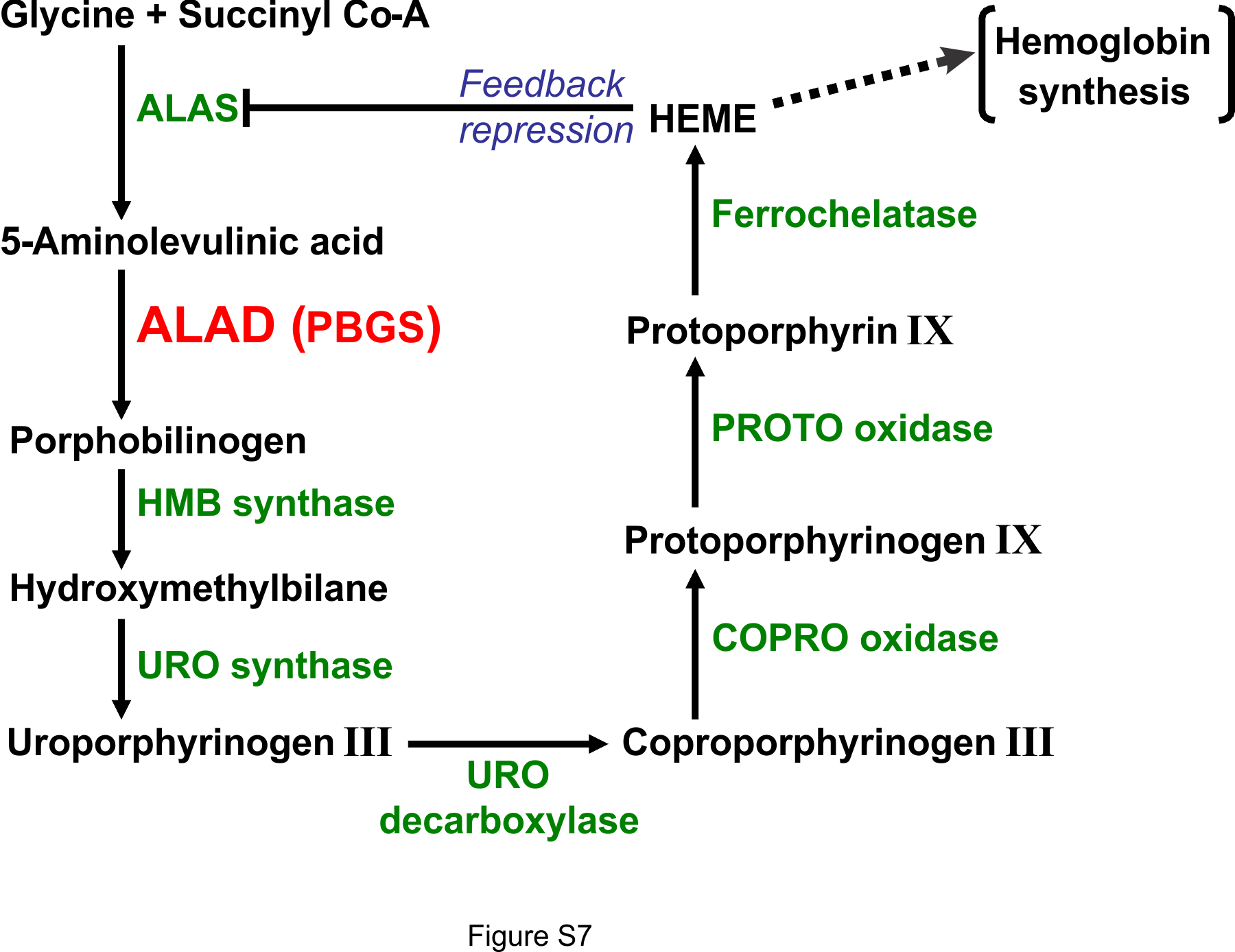

Supplement: Figure S7 — Illustration of the hemoglobin biosynthetic pathway, showing feedback repression of ALAS by heme. (Heme ultimately binds with one of several forms of globin to make hemoglobin.) Succinyl Co-A, combination of succinic acid and coenzyme-A. ALAS, 5-aminolevulinic acid synthase. ALAD, 5-aminolevulinic acid dehydratase. PBGS, porphobilinogen synthase (trivial name). HMB, hydroxymethylbilane. URO, uroporphyrinogen. COPRO, coproporphyrinogen. PROTO, protoporphyrinogen. (TIF) [file pone.0036055.s007.tif]
