# Supplementary material for: ZIP8 Zinc Transporter: Indispensable Role for Both Multiple-Organ Organogenesis and Hematopoiesis In Utero
Source: PLoS One. 2012 May 1;7(5):e36055. doi: 10.1371/journal.pone.0036055 (PMC3341399; doi:10.1371/journal.pone.0036055)
Supplement: Table S1 — Primers used for qRT-PCR. (DOC) [file pone.0036055.s008.doc]

**ZIP8 Zinc Transporter: Indispensable Role for Both Multiple-Organ**

**Organogenesis and Hematopoiesis in utero**

**Marina Gálvez-Peralta, Lei He, Lucia F. Jorge-Nebert, Bin Wang,**

**Marian L. Miller, Brian L. Eppert, Scott Afton,** and **Daniel W. Nebert**

**Table S1.** Primers used for qRT-PCR

| **mRNA** | **Forward primer** | **Reverse Primer** |
| --- | --- | --- |
| **ZIP8** | 5'-CTCGCCTTCAGTGAGGATGT-3' | 5'-GCTTTGCGTTGTGCTTTCTT-3' |
| **GAPDH** | 5'-CATCCGTAAAGACCTCTATGCC-3' | 5'-ACGCAGCTCAGTAACAGTCC-3' |
