# Supplementary material for: ZIP8 Zinc Transporter: Indispensable Role for Both Multiple-Organ Organogenesis and Hematopoiesis In Utero
Source: PLoS One. 2012 May 1;7(5):e36055. doi: 10.1371/journal.pone.0036055 (PMC3341399; doi:10.1371/journal.pone.0036055)
Supplement: Table S2 — Primers used to test ZIP8 mRNA (transcript) fragment sizes between the Slc39a8(+) and Slc39a8(neo) alleles. (DOC) [file pone.0036055.s009.doc]

**ZIP8 Zinc Transporter: Indispensable Role for Both Multiple-Organ**

**Organogenesis and Hematopoiesis in utero**

**Marina Gálvez-Peralta, Lei He, Lucia F. Jorge-Nebert, Bin Wang,**

**Marian L. Miller, Brian L. Eppert, Scott Afton,** and **Daniel W. Nebert**

**Table S2.** Primers used to test ZIP8 mRNA (transcript) fragment sizes between the *Slc39a8(+)* and *Slc39a8(neo)* alleles

| **Name of primer** | **Nucleotide sequence** | **Location of amplicon in the transcript** | **Base-pair number at which primer hybridizes** | **PCR product length (bp)** |
| --- | --- | --- | --- | --- |
| ZIP8-cDNA-201-1-F | TGAGAGGCTAGCAACTTCTCCGT | 1, 5'-UTR | 13-229 | 217 |
| ZIP8-cDNA-201-1-R | TCCTCTGACTGTGCAACCCCAA | 1, 5'-UTR |  |  |
| ZIP8-cDNA-201-2-F | TGGGAGCTTCAGTCCCGACCC | 1, 5'-UTR | 128-337 | 200 |
| ZIP8-cDNA-201-2-R | AAGGGCGATAGGCAGTGTGGC | 1, 5'-UTR |  |  |
| ZIP8-cDNA-201-3-F | CTCCGCAGGAGGCTCCGAGA | 2, 5'-UTR | 295-505 | 210 |
| ZIP8-cDNA-201-3-R | GCTAGCTGATGCAGGGACCCG | 2, 5'-UTR |  |  |
| ZIP8-cDNA-201-4-F | ACCCCCACCCTAACGGCACT | 2, 5'-UTR | 447-646 | 200 |
| ZIP8-cDNA-201-4-R | GTTGGCGCCGAACACGCTCA | 2-Coding region |  |  |
| ZIP8-cDNA-201-5-F | TGTGCTGAGCGTGTTCGGCG | 2 | 622-839 | 218 |
| ZIP8-cDNA-201-5-R | AGATCGCGGGGCAGATGGCA | 3 |  |  |
| ZIP8-cDNA-201-6-F | GCCATCTGCCCCGCGATCTT | 3 | 821-1042 | 222 |
| ZIP8-cDNA-201-6-R | AGTCCCAATAGCGAGTCCCACGA | 4 |  |  |
| ZIP8-cDNA-201-7-F | TCTGGCCTCTCTCCTGGGATTG | 4 | 946-1138 | 193 |
| ZIP8-cDNA-201-7-R | ACCAAACACAGCAACTGCTTTCTCA | 5 |  |  |
| ZIP8-cDNA-201-8-F | AGCAGTTGCTGTGTTTGGTGGATT | 5 | 1120-1324 | 205 |
| ZIP8-cDNA-201-8-R | CTCAGTGACAGCGGGGTTGGC | 6 |  |  |
| ZIP8-cDNA-201-9-F | TGCTATGCCAACCCCGCTGT | 6 | 1298-1502 | 205 |
| ZIP8-cDNA-201-9-R | CCCCAATCGCCAAGCCGTCG | 7 |  |  |
| ZIP8-cDNA-201-10-F | TCACGCTTTGTGATGCCCTCCA | 7 | 1452-1661 | 210 |
| ZIP8-cDNA-201-10-R | AGCAGGAACACGCGGAGAGGA | 8 |  |  |
| ZIP8-cDNA-201-11-F | GCAGGAATGAGCACCCGGCAA | 8 | 1604-1869 | 266 |
| ZIP8-cDNA-201-11-R | GCAGTGAAGCCGGTTAACATCCC | 9 |  |  |
| ZIP8-cDNA-201-12-F | TCCAGAGATGAACGACATGCTGAGA | 9 | 1768-1947 | 180 |
| ZIP8-cDNA-201-12-R | CTTCCCGCGTTGACGTTATACTCCA | 9, 3'-UTR |  |  |
| ZIP8-cDNA-201-13-F | AACGTCAACGCGGGAAGGCA | 9, 3'-UTR | 1931-2164 | 239 |
| ZIP8-cDNA-201-13-R | CCAAAGGCCCCATACACCAGCC | 9, 3'-UTR |  |  |
| ZIP8-cDNA-201-14-F | TGCAAAGGCTGGTGTATGGGGC | 9, 3'-UTR | 2142-2473 | 332 |
| ZIP8-cDNA-201-14-R | AGGCACTGGGATCGGTCCTGC | 9, 3'-UTR |  |  |
| ZIP8-cDNA-201-15-F | CAGACAGCTCCAGTGCATGCAGGA | 9, 3'-UTR | 2435-2634 | 200 |
| ZIP8-cDNA-201-15-R | TCAGGGACCAAGCCCTGAGCC | 9, 3'-UTR |  |  |
| ZIP8-cDNA-201-16-F | GCTTGGGCTCAGGGCTTGGT | 9, 3'-UTR | 2609-2800 | 192 |
| ZIP8-cDNA-201-16-R | TCCCACCCAACATATTCTCCACCAT | 9, 3'-UTR |  |  |
| ZIP8-cDNA-201-17-F | GCCTGAGGAGTATGCGAGACTGG | 9, 3'-UTR | 2714-2916 | 203 |
| ZIP8-cDNA-201-17-R | TCTCTGACACAGCCCAGCCCA | 9, 3'-UTR |  |  |
| ZIP8-cDNA-201-18-F | CTGGGCTGGGCTGTGTCAGA | 9, 3'-UTR | 2895-3104 | 210 |
| ZIP8-cDNA-201-18-R | CAGACTGATCGTCTACAACGTGCTC | 9, 3'-UTR |  |  |
| ZIP8-cDNA-201-19-F | GCTCACTGAGTGTTTGAGCACGTT | 9, 3'-UTR | 3065-3430 | 370 |
| ZIP8-cDNA-201-19-R | TGCCTCTCTTCTCATAACACCAGCC | 9, 3'-UTR |  |  |
| ZIP8-cDNA-001-1-F | AGCTAGCCCACCAACTTGCGAC | 1, 5'-UTR | 4-230 | 230 |
| ZIP8-cDNA-001-1-R | CCGCTGTGGTGCCTACTCCCT | 1, 5'-UTR |  |  |
| ZIP8-cDNA-001-2-F | ACACCAGGAAGGGGGCTAGGG | 2, 5'-UTR | 169-379 | 211 |
| ZIP8-cDNA-001-2-R | TGCCTGGCCCAGTTAGCGAGA | 2, 5'-UTR |  |  |
| ZIP8-cDNA-003-1-F | GGGCTTCAGCAAACCAGGCTCC | 1, 5'-UTR | 3-263 | 263 |
| ZIP8-cDNA-003-1-R | TAGCGAGACACTCCGTGCCCC | 1, 5'-UTR |  |  |
