# Supplementary material for: ZIP8 Zinc Transporter: Indispensable Role for Both Multiple-Organ Organogenesis and Hematopoiesis In Utero
Source: PLoS One. 2012 May 1;7(5):e36055. doi: 10.1371/journal.pone.0036055 (PMC3341399; doi:10.1371/journal.pone.0036055)
Supplement: Table S3 — Comparison of blood chemistry tests among the three genotypes at age of PND1. (DOC) [file pone.0036055.s010.doc]

**ZIP8 Zinc Transporter: Indispensable Role for Both Multiple-Organ**

**Organogenesis and Hematopoiesis in utero**

**Marina Gálvez-Peralta, Lei He, Lucia F. Jorge-Nebert, Bin Wang,**

**Marian L. Miller, Brian L. Eppert, Scott Afton,** and **Daniel W. Nebert**

**Table S3.** Comparison of blood chemistry tests among the three genotypes at age of PND1

|  | **Total iron** | **TIBC** | **ALT** | **AST** | **HDL** | **LDL** | **Cholesterol** | **TGs** |
| --- | --- | --- | --- | --- | --- | --- | --- | --- |
|  | **(μg/dL)** | | **(U./L)** | | **(mg/dL)** | | | |
| ***Slc39a8(+/+)*** | 204 | 521 | 14 | 291 | 30 | 25 | 50 | 129 |
| ***Slc39a8(+/neo)*** | 268 | 446 | 16 | 296 | 25 | 26 | 60 | 266 |
| ***Slc39a8(neo/neo)*** | **<5.0** | **<8** | **<6.0** | **<20** | 33 | 34 | 64 | **8.0** |

Strikingly diminished values are denoted in **red**.

Total bilirubin was <0.1 mg/dL in all three genotypes.

Ferritin and ferritin saturation tests were not possible to measure, because the immunogenic specificity of the test was for the human protein.

Total numbers of pups from all pooled blood included**:** 26 *Slc39a8(+/+)***;** 35 *Slc39a8(+/neo)***;** and 17 *Slc39a8(neo/neo)* pups.
