# Supplementary material for: ZIP8 Zinc Transporter: Indispensable Role for Both Multiple-Organ Organogenesis and Hematopoiesis In Utero
Source: PLoS One. 2012 May 1;7(5):e36055. doi: 10.1371/journal.pone.0036055 (PMC3341399; doi:10.1371/journal.pone.0036055)
Supplement: Data S1 — All Materials and Methods are described in detail. In addition, P-values for all comparisons of data in Figures 2 & 8 are provided. Finally, seven supplemental figures and three supplemental tables are included. (DOC) [file pone.0036055.s011.doc]

**SUPPLEMENTARY DATA ONLINE**

**ZIP8 Zinc Transporter: Indispensable Role for Both Multiple-Organ**

**Organogenesis and Hematopoiesis in utero**

**Marina Gálvez-Peralta^1^, Lei He^1,a^, Lucia F. Jorge-Nebert^1^, Bin Wang^1,b^,**

**Marian L. Miller^1^, Brian L. Eppert^1^, Scott Afton^2^,** and **Daniel W. Nebert^1^,***

**^1^***Department of Environmental Health, and Center for Environmental Genetics (****CEG****)*

*University of Cincinnati Medical Center, Cincinnati, Ohio 45267-0056*

**^2^***Department of Chemistry, University Cincinnati School of Arts and Sciences*

*Cincinnati OH 45221*

**^a^**Current address**:** *Mass. General Hospital Cancer Center, Harvard Medical School, Boston, MA 02114*

**^b^**Current address**:** *Department of Pediatric Surgery, Cincinnati Children's Hospital Medical Center, Cincinnati, OH 45229*

*****Email**:** [dan.nebert@uc.edu](mailto:dan.nebert@uc.edu)

**Key Words:** zinc**;** iron**;** cadmium**;** solute carrier (***Slc***) gene superfamily**;** ZIP transporter gene family**;** ***Slc39*** gene family**;** ZIP8 Zn^2+^/(HCO_3_^–^)_2_ symporter**;** erythropoiesis in utero**;** multiple-organ development in utero**;** hypomorphic mouse model

**Materials and Methods**

**Animals**

The *Slc39a8(neo)* allele was present in a "mixed background", *i.e.* approximately 50% C57BL/6J and 50% 129/SvJ. When the *Slc39a8(neo)* allele was bred into the C57BL/6J background (*i.e.* eight crosses giving >99.8% C57BL/6J genetic background), the *Slc39a8(neo/neo)* defective phenotype was more pronounced (*e.g.* more deaths at GD18.5), and comparisons of the phenotype in the two genetic backgrounds will be the subject of a future manuscript.

In the rescue experiment, *Slc39a8(+/neo)* mice were crossed with our previously created *BTZIP8-3* BAC-transgenic mouse line [1]**;** this line carries normal diploid copies of the C57BL/6J *Slc39a8* gene on chromosome 3, plus three copies of BAC DNA containing the *Slc39a8* gene (of 129S6/SvEvTac origin) inserted elsewhere in the genome. This *BTZIP8-3* line is >99.8% C57BL/6J genetic background. All mouse experiments were conducted in accordance with the National Institutes of Health standards for the care and use of experimental animals and the University Cincinnati Medical Center Institutional Animal Care and Use Committee.

**Total RNA Preparation**

Using Tri-Reagent (TR18, Molecular Research Center, Inc.; Cincinnati, OH), we isolated total RNA (combining those of the same genotype from one litter) from individual embryos, fetuses, yolk sacs, placentas, and PND1 organs (liver, lung, kidney, heart, spleen, thymus, cerebrum, cerebellum, and carcass). We also isolated total RNA from mouse GD14.5 fetal fibroblast (**MFF**) cells and fetal liver-derived cultures. Total RNA from GD17.5 fetal liver was isolated using Precellys ceramic-bead kits (1.4-mm) (Cayman Chemical Company; Ann Arbor, MI). Tri-Reagent lysis buffer was supplemented with 0.002% linear acrylamide (5 mg/mL) (Amresco; Solon, OH). In each case, we followed the manufacturer’s recommendations.

**Reverse Transcription and Quantitative Real-Time PCR (qRT-PCR) Analysis**

Total RNA (2.5 µg), from everything described above, was used as a template for reverse transcription and primed with oligo(dT), using the SuperScript III first-strand kit, following the manufacturer's recommendations (Invitrogen). Total RNA (2.5 μg) was added to reactions containing 3.8 μM oligo(dT)_20_ and 0.77 mM dNTP, to a final volume of 13 μL. Reactions were incubated at 65^o^C for 5 min, then 4^o^C for 2 min. Next, we added 7 μL of a solution containing 14 mM dithiothreitol and 40 units of RNaseOUT Recombinant RNase inhibitor^TM^ (Verso cDNA kit, AB-1453/B, Thermo Scientific; Waltham, MA). After incubation at room temperature for 2 min, 1 μL of RT enzyme was added to each sample. Reaction tubes were incubated at 42^o^C for 30 min, followed by 75^o^C for 10 min (to inactivate reverse transcriptase) and placed immediately on ice. Diethylpyrocarbonate (**DEPC**)-treated distilled water (80 μL) was added to dilute the cDNA that had been generated, and the resultant mixture was stored at –80^o^C until use.

We performed qRT-PCR in the Bio-Rad DNA Engine Opticon 2^TM^ (Bio-Rad Laboratories; Hercules, CA), using iQ SYBR Green Supermix^TM^ (170-8882, Bio-Rad Laboratories). The housekeeping gene glyceraldehyde-3-phosphate dehydrogenase (**GAPDH**) mRNA was employed as the internal control. A standard curve using serial dilutions of total RNA resulted in excellent linearity (*r* = 0.99), indicating our qRT-PCR results were valid. Primers used are listed in **Table S1**. The *Slc39a8(+/+)* wild-type ZIP8 mRNA/GAPDH mRNA ratio (left-most in each panel) and mRNA or protein expression levels were expressed as linear fold-changes––relative to the normalized wild-type control.

Statistical analyses of **Fig. 2** data are as follows. For **total embryo: GD11.5,** *(+/+)* vs (*neo/neo)* *P* <0.001 and *(+/neo)* vs (*neo/neo)* *P* <0.001**;** **GD13.5,** *(+/+)* vs (*neo/neo)* *P* <0.001 and *(+/+)* vs (*+/neo)* *P* = 0.004. For **total fetus: GD16.5,** *(+/+)* vs (*neo/neo)* *P* <0.001 and *(+/+)* vs (*+/neo)* *P* <0.001. For **yolk** **sac: GD11.5,** *(+/+)* vs (*neo/neo)* *P* < 0.001 and *(+/neo)* vs *(neo/neo)* *P =*0.049**;**  **GD13.5,** *(+/+)* vs (*neo/neo)* *P* = 0.036 and *(+/+)* vs *(+/neo)* *P =*0.039. For **placenta: GD11.5,** *(+/+)* vs (*neo/neo)* *P* = 0.049**;** **GD13.5,** *(+/+)* vs (*neo/neo)* *P* <0.001 and *(+/+)* vs *(+/neo)* *P* <0.001. For **PND1** **liver:** *(+/+)* vs (*neo/neo)* *P* = 0.011 and *(+/neo)* vs (*neo/neo)* *P*= 0.027. For **PND1** **lung:** *(+/+)* vs (*neo/neo)* *P* = 0.021 and *(+/+)* vs *(+/neo)* *P =* 0.010. For **PND1** **kidney:** *(+/+)* vs (*neo/neo)* *P* <0.001 and *(+/+)* vs *(+/neo)* *P* <0.001. For **PND1** **cerebrum:** *(+/+)* vs (*neo/neo)* *P* = 0.045. For **PND1** **cerebellum:** *(+/+)* vs (*neo/neo)* *P* = 0.00014. For **PND1** **thymus:** *(+/+)* vs (*neo/neo)* *P*  <0.001. For **PND1** **heart:** *(+/+)* vs (*neo/neo)* *P* = 0.001 and *(+/+)* vs *(+/neo)* *P =*0.003. For **PND1 carcass:** *(+/+)* vs (*neo/neo)* *P* = 0.007 and *(+/neo)* vs (*neo/neo)* *P*= 0.025.

**Determination of mRNA Fragment Sizes by PCR**

To determine whether ZIP8 mRNA size might differ among the three genotypes, we designed overlapping PCR reactions that cover the 5' UTR, coding region and 3' UTR regions. The mRNA sequence was obtained from the database ENSEMBL. Analysis of ZIP8 mRNA also included all three alternative exons 1 (denoted 001, 003 and 201), previously described in detail [2]. RNA isolation from PND1 kidneys of *Slc39a8(+/+)*, *Slc39a8(+/neo)* and *Slc39a8(neo/neo)* pups was carried out, using the Precellys beads system**;** formation of cDNA from total RNA is described above. The cDNA was quantified using a NanoDrop *ND1000* Spectrophotometer (Thermo; Wilmington, DE), and equal amounts of cDNA were used for each PCR reaction. PCR reactions were performed with GoTaq Hot Start Green Master Mix (Promega; Madison, WI) and the Biorad C1000^TM^ thermal cycler (Hercules, CA). Reactions were run on 2% agarose gels. Primers, as well as the predicted PCR fragment size and mRNA segment covered, are listed in **Table S2**.

**Western Immunoblot Analysis**

Tissue extracts were prepared using T-PER tissue protein extraction reagent (Thermo) supplemented with phenylmethylsulfonyl fluoride (**PMSF**; final concentration 1 mM), leupeptin (1 μg/mL) and aprotinin (2 μg/mL) (Sigma-Aldrich; St. Louis, MO). We tested other lysis buffers (*e.g.* triton or sodium dodecylsulfate), but the T-PER buffer gave the best results for ZIP8 protein detection. Protein concentrations were determined using the Thermo BCA protein reagent (Thermo Scientific; Rockford, IL). Tissue extracts (10-40 μg protein per sample) were subjected to SDS-PAGE electrophoresis [3] on 12% polyacrylamide mini-gels at 125 mV per gel under denaturing conditions. Separated proteins were transferred to nitrocellulose (90 V; 1 h) and visualized with Ponceau S to verify equivalent loading across lanes. Mouse monoclonal β-actin (**ACTB**) (Sigma-Aldrich; St. Louis, MO) antibody was used as a lane-loading control. Western blot analysis was performed using rabbit polyclonal anti-human ZIP8 (kind gift of Daren Knoell). Horseradish peroxidase-conjugated secondary antibodies (Santa Cruz, Biotechnology, Inc.; Santa Cruz, CA) and Super Signal West Pico Chemiluminescent Substrate**^TM^** (Thermo Scientific) were used for visualization**;** exposure times ranged from 1 min to 6 h. Densitometric analyses of the Western immunoblot lanes were performed using ImageJ software (<http://rsb.info.nih.gov/ij/>). The *Slc39a8(+/+)* wild-type ZIP8 protein/ACTB protein ratio (left-most in each panel) and protein expression levels were expressed as linear fold-changes––relative to the normalized wild-type control for each organ.

**Histology and Analysis of Hematopoiesis**

The tissues shown in **Table 2** and **Fig. 2** were harvested, put into 10% neutral buffered formalin, or cut into 2-mm pieces with a razor blade and fixed in phosphate-buffered 4% paraformaldehyde. Following formalin fixation and dehydration, the tissues were embedded in paraffin, sectioned, and the slides were stained with hematoxylin and eosin and photographed at several magnifications, using standard procedures. Paraformaldehyde-fixed, ethanol- and propylene dioxide-dehydrated tissues were embedded in Spurr's resin, cut at 2-micron thickness, and stained with Toluidine blue O. At PND1, the pups were decapitated and whole blood was collected and analyzed by Hemavet HV950FS multi-species hematology instruments (Drew Scientific, Inc.; Oxford, CT). Blood smears from GD16.5 fetuses onward through PND1 pups were obtained from peripheral blood collection with a heparinized microcapillary tube**;** after methanol fixation, slides were stained with Giemsa.

**Fetal and Neonatal Blood for Analysis**

Peripheral blood smears from fetuses and PND1 pups, were made from a drop of blood (20-25 μL) taken while opening the neck-thorax to extract thymus, heart and lungs (*i.e.* this is circulating blood), after which we carried out air drying, methanol fixation, and staining with Wright-Giemsa. In the case of serum chemistry tests, the head of the PND1 pup was cut off and blood was collected from the open area. Following centrifugation of the blood, serum was collected and stored at –80^o^C until sufficient amounts could be analyzed for the various blood chemistry tests.

**Blood Chemistry Tests**

In order to obtain a sufficient volume for these tests, we isolated and pooled blood (twice) from whole PND1 newborns. Total numbers of pups from all pooled blood included**:** 26 *Slc39a8(+/+)***;** 35 *Slc39a8(+/neo)***;** and 17 *Slc39a8(neo/neo)* pups. Tests performed at the Cincinnati Children's Hospital Medical Center Central Laboratory included serum iron, total iron binding capacity (**TIBC**), total bilirubin, alanine transaminase (**ALT**), aspartate transaminase (**AST**), high-density lipoprotein (**HDL**-cholesterol), low-density lipoprotein (**LDL**-cholesterol), and triglycerides (**TGs**). Ferritin and transferrin saturation tests could not be performed in this lab because the immunoassay for ferritin is specific for human protein.

**Flow Cytometry**

Individual cells from GD13.5 yolk sacs and GD16.5 livers were mechanically dissociated by pushing with a syringe through a 20-gauge needle and pressing the suspension through a 100-μm cell strainer in 5 mL of RPMI 1640 medium. The cells were then harvested, washed, resuspended in FACS buffer (1x PBS, 0.5% bovine serum albumin, 2 mM EDTA, and 0.05% NaN_3_), and counted. Antigen-antibody surface reactions for erythroid, myeloid and lymphoid cells were carried out on ice for 30 min. Anti-mouse antibodies included**:** antibody clone PE-TER119 for Ly-76 (0.25 μg/10^6^ cells)**;** antibody clone R17217 for FITC-CD71 (0.5 μg/10^6^ cells)**;** antibody clone M1/70 for Alexa Fluor 647-CD11b (0.125 μg/10^6^ cells)**;** and antibody clone 145-2C11 for PE-Cy5-CD3e (0.25 μg/10^6^ cells). All antibodies were acquired from eBioscience (San Diego, CA). After being washed, cells were resuspended in FACS buffer, and flow cytometry was performed using a FACSCalibur (BD Biosciences; San Jose, CA). The data were analyzed using the FlowJo software (Tree Star; Ashland, OR). Positive staining thresholds were determined from appropriate epitope control staining. Erythroid differential stages were determined by studying the cell distribution of PE-Ter119 and FITC-CD71 positive cells.

**Cell Cultures**

GD14.5 fetal livers, plus mouse fetal fibroblasts (**MFFs**) from the remainder of the fetus minus the liver, were generated from *Slc39a8(+/+)*, *Slc39a8(+/neo)* and *Slc39a8(neo/neo)* mice**;** these were cultured at 37°C in 5% CO_2_ in Dulbecco’s modified Eagle’s medium (**DMEM**) (Invitrogen; Carlsbad, CA) plus 10% fetal bovine serum (Hyclone; Logan, UT). The MFF and fetal liver cultures were used for ZIP8 mRNA quantification (**Fig. 4**) and divalent metal cation uptake experiments (**Fig. 7**). Additional details of these procedures have previously been described [4;5].

**Uptake of Zn^2+^, Cd^2+^ and Fe^2+^ in MFF or Fetal Liver-Derived Cultures**

Divalent zinc, cadmium and iron were purchased as chloride salts from Fisher Scientific (Pittsburgh, PA), divalent Zn from Sigma (St. Louis, MO). ^65^ZnCl_2_ [4.28 mCi/mg in 0.1 M HCl] and ^109^CdCl_2_ [159.42 mCi/mg in 0.1 M HCl] were bought from Perkin Elmer (Waltham, MA), and ^55^FeCl_2_ [120 mCi/mg in 0.1 M HCl] from National Laboratory of Oak Ridge (Oak Ridge, TN)**;** [1 mCi = 37 mBq]. For uptake medium we used a modified version of Hanks’ balanced salt solution (**HBSS**) (Invitrogen; Carlsbad, CA), previously described [4]. In the case of Fe uptake, HBSS medium was supplemented with 1 mM ascorbic acid, in order to maintain Fe^2+^ as the reduced divalent cation.

All procedures for measuring radiolabeled metal cation uptake in cultured cells have previously been detailed [2;4;5]. Incubation time was 20 min. MFF and fetal liver cell cultures were derived from GD14.5 fetuses, as described earlier.

**Sample Preparation for Determining Tissue Zn, Fe, Cu and Mn Content**

On PND1, *Slc39a8(+/+)*, *Slc39a8(+/neo)* and *Slc39a8(neo/neo)* tissues (~100 mg of liver, lung, kidney, and heart) were placed in separate septum-sealed glass tubes and treated with 0.5 mL of HNO_3_ and subjected to microwave acid digestion (150 W; 1.00-min ramp time, 2.00-min hold time; 170°C), using the CEM Explorer system equipped with the Discover auto-sampler (Matthews, NC) [6]. Digested samples were then diluted to 10.0 mL with doubly-distilled water.

**Inductively-Coupled Plasma Mass Spectrometry (ICP-MS)**

An Agilent 7500ce (Agilent Technologies; Santa Clara, CA) ICP-MS––equipped with shielded-torch and collision/reaction-cell technology––was used for the element-specific detection of Zn, Fe, Cu and Mn. The collision/reaction cell consisted of an octopole-ion guide, operated in “rf-only” mode, and also served for the removal of polyatomic interferences. Forward power was 1500 W (with shielded torch)**;** plasma gas flow rate was 15.0 L min^-1^**;** auxiliary gas flow rate was 1.0 L min^-1^**;** carrier gas flow rate was 1.00 L min^-1^**;** nebulizer was glass-expansion microcentric**;** spray chamber was ~2°C (Scott double-channel)**;** sampling depth was 7 mm**;** sampling and skimmer cones were nickel**;** dwell time was 0.1 s**;** isotopes that we monitored included ^66,68^Zn, ^57^Fe, ^63^Cu and ^55^Mn**;** the octopole reaction system used helium (the flow was optimized prior to each experiment).

Statistical analysis of data (ng metal per mg tissue) in **Fig. 8** is as follows. For **zinc in liver**, all-pairwise analysis of variance *P* = 0.007**;** *Slc39a8(+/+)* vs *(neo/neo)* *P* = 0.003 and *(+/neo)* vs *(neo/neo)* *P* = 0.02. For **iron in liver**, all-pairwise analysis of variance *P* = 0.006**;** *(+/+)* vs *(neo/neo)* *P* <0.001 and *(+/neo)* vs *(neo/neo)* *P* = 0.006. For **zinc in lung**, no significant differences. For **iron in lung**, all-pairwise analysis of variance *P* = 0.049**;** *(+/+)* vs *(neo/neo)* *P* = 0.021 and *(+/+)* vs *(+/neo)* *P* = 0.047. For **zinc in kidney**, no significant differences. For **iron in kidney**, *(+/+)* vs *(+/neo)* *P* = 0.024. For **zinc in heart**, *(+/+)* vs *(neo/neo)* *P* = 0.009 and *(+/+)* vs *(+/neo)* *P* = 0.16. For **iron in heart**, no significant differences.

**5-Aminolevulinic Acid Dehydratase (ALAD) Enzyme Assay**

The ALAD assay was performed as described [7-9] with modifications. The substrate 5-aminolevulinic acid (**ALA**) is added to homogenates [120 mg liver homogenized in 240 μL cold 0.15 N KCl (1**:**2 or 0.333 mg/mL)] and incubated to produce porphobilinogen (**PBG**). PBG is a pyrrole-containing structure detected by generating its adduct with *p*-dimethylaminobenzaldehyde (Ehrlich’s reagent) in strongly acidic medium**;** the adduct is pink-purple in color, measured spectrophotometrically at 555 nm.

Depending on genotype, either two or three PND1 livers were pooled to reach the desired liver weight. If required, the liver homogenate can be stored at –80°C. Reagents included**:** β-mercaptoethanol (Sigma; St. Louis, MO), 25 mM in 0.1 M *bis*-tris-propane (**BTP**) buffer, pH 6.7, prepared daily**;** ALA (Research Products Intl.; Mt. Prospect, IL), 0.05 M in 0.1 M BTP buffer, adjusted to pH 6.7 by adding 10 M NaOH, prepared once per week (stored at 4°C)**;** HgCl_2_ (Fisher Sci.; Fair Lawn, NJ) 0.1 M in 10% trichloroacetic acid (**TCA**)**;** modified Ehrlich’s reagent (prepared daily), 200 mg of American Chemical Society reagent-grade *p*-dimethylaminobenzaldehyde (Acros; Geel, Belgium), dissolved in 6 mL glacial acetic acid, followed by the slow, careful addition of 3.2 mL 70% perchloric acid, with sufficient acetic acid added to make a final volume of 10 mL**;** PBG standard (Frontier Sci.; Logan, UT), 0.819 mM in 0.1 M BTP buffer, pH 6.7**;** and ZnCl_2_ (Fisher Sci.), 1.56 mM in 0.1 M BTP buffer, pH 6.7. The 0.1 M BTP buffer (pH 6.7) and the ALA solution were first passed through a column packed with Chelex-100 resin, 200-400 mesh, sodium form (Bio-Rad; Hercules, CA) to eliminate traces of divalent cations (*e.g.* lead, mercury, and especially zinc) which could affect enzyme activity during the assay.

Liver homogenate (25 μL, equivalent to 8.33 mg) was added to a 1.7-mL microfuge containing 50 μL β-mercaptoethanol in BIP buffer. Following pre-incubation of the mixture under argon at 37°C for 15 min, ALA in BIP buffer (50 μL) was added to make 10 mM β-mercaptoethanol and 20 mM ALA in the final reaction mixture (125 μL)**;** this was incubated at 37°C for 15 min. The reaction was stopped by adding the 0.1 M HgCl_2_ solution. The precipitate was centrifuged (13,000 rpm for 5 min)**;** the supernatant (200 μL) was diluted with 5% TCA (100 μL), followed by addition of 300 μL modified Ehrlich’s reagent. After allowing the color to develop for 5 min, the samples were read at 475 nm. Specific activity is defined as nmol PBG produced/min/mg protein at 37°C. Protein determinations were carried out by the bicinchoninic acid (**BCA**) assay (Thermo Scientific; Rockford, IL).

Each liver homogenate was tested in three assays simultaneously**:** (i) non-activated (native enzyme is used) in which the above-described procedure is applied without modifications**;** (ii) zinc-activated, in which 1.5 μL ZnCl_2_ is added before the reaction, making a final added zinc concentration of 18.1 μM**;** (iii) blank assay in which HgCl_2_ is added during pre-incubation, prior to addition of the ALA substrate. Any positive absorbance recorded in the blank was subtracted from the absorbance of the non-activated and activated assays. The zinc-activated vs non-activated results (amount of PBG formed) were translated into specific activities, which in turn were used to calculate the Relative Index (**R.I.**) of ALAD activity.

Quantification was achieved using a calibration curve**;** PBG standard solutions (from 0 to 98.3 μM) were added to the enzyme reaction mixture (liver homogenate plus β-mercaptoethanol, in absence of the substrate ALA) in such a way that the final volume of the mixture was unaffected (more 0.1 M BTP buffer was added, as needed, to complete the final volume of 125 μL).

**Statistical Analyses**

For quantitative histology, we generated means and standard-errors-of-the-means (S.E.M.) with Excel or the General Linear Model of SAS©. We used Vd (volume density) to determine the density of hematopoietic islands (relative to total number of all cell types) in GD13.5 yolk sac and GD16.5 liver. For all measurements, all experiments were confirmed at least in triplicate. Calculations were performed using SPSS version 13.0 (Microsoft; Somers, NY). Data were normally distributed and are presented as means + S.E.M. All *P*-values of <0.05 are regarded as statistically significant.

**Supplementary References**

1. Wang B, Schneider SN, Dragin N, Girijashanker K, Dalton TP, et al. (2007) Enhanced cadmium-induced testicular necrosis and renal proximal tubule damage caused by gene-dose increase in a *Slc39a8*-transgenic mouse line. Am J Physiol Cell Physiol 292: C1523–C1535.

2. Girijashanker K, He L, Soleimani M, Reed JM, Li H, et al. (2008) *Slc39a14* gene encodes ZIP14, a metal/bicarbonate symporter**:** similarities to the ZIP8 transporter. Mol Pharmacol 73: 1413–1423, 2008.

3. Brown EG (1988) Mixed anionic detergent/aliphatic alcohol-polyacrylamide gel electrophoresis alters the separation of proteins relative to conventional sodium dodecyl sulfate-polyacrylamide gel electrophoresis. Anal Biochem 174: 337–348.

4. He L, Girijashanker K, Dalton TP, Reed J, Li H, et al. (2006) ZIP8, member of the solute-carrier-39 (*SLC39*) metal-transporter family**:** characterization of transporter properties. Mol Pharmacol 70: 171–180.

5. Dalton TP, He L, Wang B, Miller ML, Jin L, et al. (2005) Identification of mouse SLC39A8 as the transporter responsible for cadmium-induced toxicity in the testis. Proc Natl Acad Sci USA 102: 3401–3406.

6. Afton SE, Caruso JA (2009) Effect of Se antagonism on the metabolic fate of Hg in *Allium fistulosum*. J Anal At Spectrom 24: 759–766.

7. Mauzerall D, Granick S (1956) The occurrence and determination of δ-amino-levulinic acid and porphobilinogen in urine. J Biol Chem 219: 435-446, 1956.

8. Russell RL, Coleman DL (1963) Genetic control of hepatic δ-aminolevulinate dehydratase in mice. Genetics 48: 1033–1039.

9. Tang L, Stith L, Jaffe EK (2005) Substrate-induced interconversion of protein quaternary structure isoforms. J Biol Chem 280: 15786–15793.
